# New Dual Pan-PI3K/mTOR Inhibitor: Design, Synthesis, Cytotoxic Action, Permeation, Metabolic Stability, and In Silico Protein–Ligand Interaction

**DOI:** 10.1021/acsomega.5c10162

**Published:** 2026-02-05

**Authors:** Cristiane Aparecida e Silva, Raysa Magali Pillpe-Meza, Wesley Leandro Gouveia, Joana D’Arc da Silva Trindade, Gisele Barbosa, Amanda Marques Seixas Vieira, Heber Victor Tolomeu, Rayane França Pereira, Carlos Antônio do Nascimento Santos, Leonardo Freire-de-Lima, Lidia Moreira Lima

**Affiliations:** † Laboratório de Avaliação E Síntese de Substâncias Bioativas (LASSBio), Instituto de Ciências Biomédicas, 28125 Universidade Federal do Rio de Janeiro, Avenida Carlos Chagas Filho, 373, Cidade Universitária, Rio de Janeiro, Rio de Janeiro CEP 21941-902, Brasil; ‡ Programa de Pós-Graduação Em Farmacologia E Química Medicinal, Instituto de Ciências Biomédicas, Universidade Federal do Rio de Janeiro, Avenida Carlos Chagas Filho, 373, Cidade Universitária, Rio de Janeiro, Rio de Janeiro CEP 21941-902, Brasil; § Laboratório de Glicobiologia (LABGLICO), Instituto de Biofísica Carlos Chagas Filho, 28125 Universidade Federal do Rio de Janeiro, Avenida Carlos Chagas Filho, 373, Cidade Universitária, Rio de Janeiro, Rio de Janeiro CEP 21941-902, Brasil

## Abstract

The PI3K/AKT/mTOR pathway is frequently dysregulated in cancer, contributing to tumor progression, drug resistance, and poor prognosis. Dual PI3K/mTOR inhibitors such as gedatolisib have shown clinical promise, but they still face challenges, including low solubility, poor metabolic stability, and limited activity against resistant tumor phenotypes. Here, we report a proof-of-concept study exploring structural modifications on compound **5f**, a simplified gedatolisib analog, to generate a novel small subseries of morpholino-triazine derivatives (**9a**–**f**). The goal was to improve molecular interactions within the affinity site of PI3K, investigate the impact on isoform selectivity, and evaluate pharmacological properties relevant to early optimization. Among these, compound **9a** (LASSBio-2337) emerged as a dual pan-PI3K/mTOR inhibitor (IC_50_: 0.3–5.8 μM), showing cytotoxic effects in leukemia cell lines (CC_50_: 4.37–9.44 μM), including those with multidrug resistance (Lucena, MDR phenotype), while sparing nontumor hPBMCs. Although aqueous insoluble, **9a** displayed moderate PAMPA-GIT permeability and low metabolic stability in rat liver microsomes, underscoring its potential as a lead for further optimization. This integrated study provides structural, mechanistic, and pharmacokinetic insights to guide next-generation PI3K/mTOR inhibitor design.

## Graphical abstract

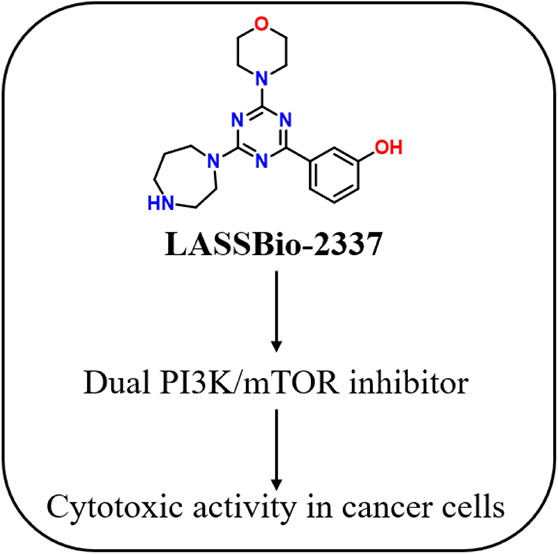

## 1 Introduction

PI3K and mTOR have been identified as promising targets for cancer treatment. They participate in related signaling networks to transmit cellular growth and survival signals that are hallmarks of tumor growth.^1–3^ PI3K is responsible for the phosphorylation of the 3′–OH moiety on the inositol ring phosphatidylinositol (4,5) bis-phosphate (PtdIns­(4,5)­P_2_ or PIP_2_) to phosphatidylinositol (3,4,5) tris-phosphate (PtdIns­(3,4,5)­P_3_ or PIP_3_). PIP3 serves as a docking site for proteins containing pleckstrin homology (PH) domains, such as AKT, leading to its activation.^1,4,5^ PI3Ks are divided into three classes (I, II, and III), with class I being the most studied due to its involvement with tumorigenesis, cell proliferation, growth, and survival. Class IA PI3Ks (PI3Kα, PI3Kβ, and PI3Kδ) consist of a catalytic subunit (p110α, p110β, and p110δ, respectively) and a p85 regulatory subunit. The class IB subtype (PI3Kγ) combines a catalytic p110γ subunit with a regulatory p101.^5–8^ mTOR is an atypical serine/threonine kinase that modulates cell growth and metabolism in response to extracellular nutrient and energy factors. It is a member of the PIKK (phosphatidylinositol like kinase) family.^9–11^ The PI3K/AKT/mTOR signal transduction pathway is dysregulated in many cancers, contributing to cellular transformation and tumor growth.^1–3^ Genomic alterations in the PI3K pathway such as mutational activation of PI3Kα or dysfunction of the tumor suppressor PTEN are closely linked to the development and progression of a wide range of cancers, such as colon, breast, head and neck, and nonsmall cell lung cancers.^10,12,13^ On the other hand, mTORC1 dysregulation is often associated with the development of cancer, diabetes, and neurological diseases. Therefore, the inhibition of these key targets has great potential for cancer treatment^9,11^

Remarkable progress has been made on the design, synthesis, and evaluation of PI3K and mTOR dual inhibitors.^6,14^ PI-103 (**1**) is the first morpholine-based PI3K and mTOR dual inhibitors, resulting in the subsequent discovery of the triazine-morpholine derivatives (Figure 1) with great antitumor activity. Several promising candidates, such as ZSTK474 (**2**), PQR309 (**3**), and Gedatolisib (PF05212384) (**4**) are currently in clinical trials.^1,5,7–16^ The latter, developed by Pfizer, is under clinical phase studies for the treatment of breast, pancreatic, and head and neck cancers.^1,5,14^

**Figure 1:**
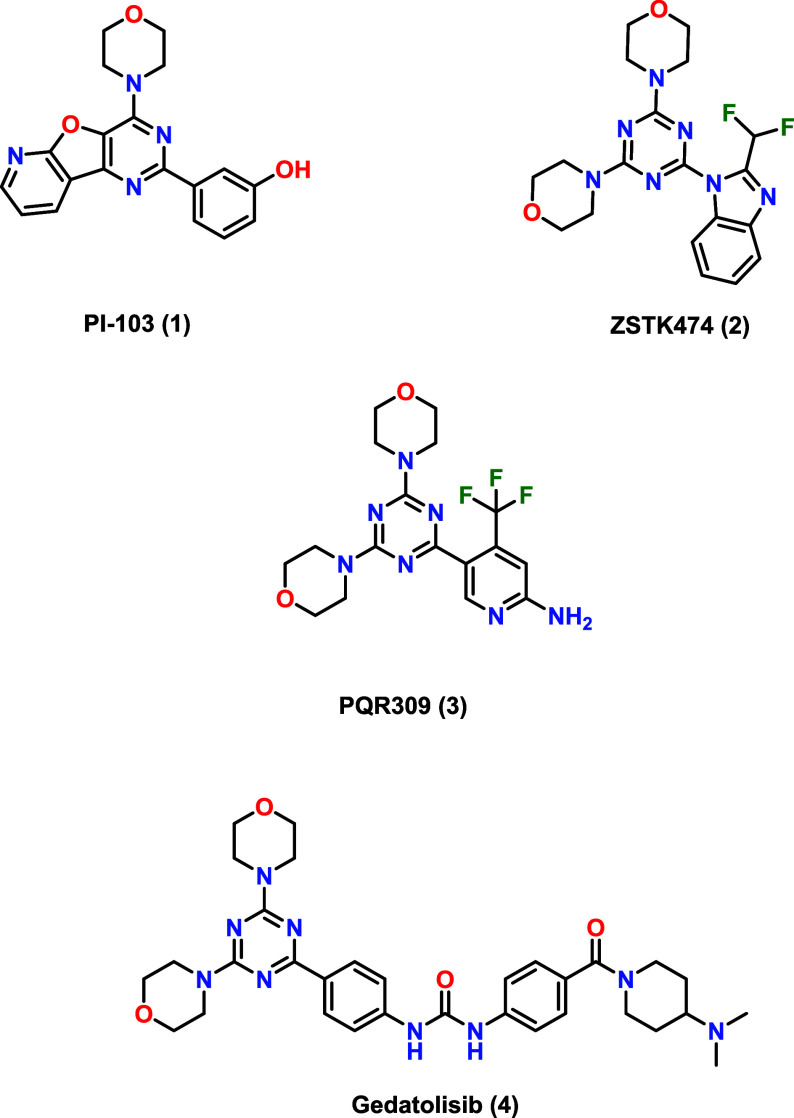
Examples of morpholino-substituted triazine PI3K/mTOR dual inhibitors.

In a recent study, we synthesized a series of simplified analogs of gedatolisib (**4**) (Figure 2), which were evaluated for their cytotoxic activity against human tumor cell lines, and the cellular mechanism of action was studied through phenotypic assays.^17^ Among the synthesized compounds, **5f** exhibited the most favorable biological effect with CC_50_ values of 62.15 and 37.04 μM in PC-3 and MCF-7 cell lines, respectively. In hematological neoplasia cell lines, **5f** showed CC_50_ values of 6.25 and 9.76 μM in CCRF-CEM and MOLT-4 cell lines, respectively.^17^ The cellular mechanism of action of **5f** was investigated, and it was demonstrated that it inhibited the phosphorylation of AKT in CCRF-CEM and MOLT-4 cell lines like gedatolisib. However, this compound exhibited 100 times less potency than the standard gedatolisib (**4**).^17^

**Figure 2:**
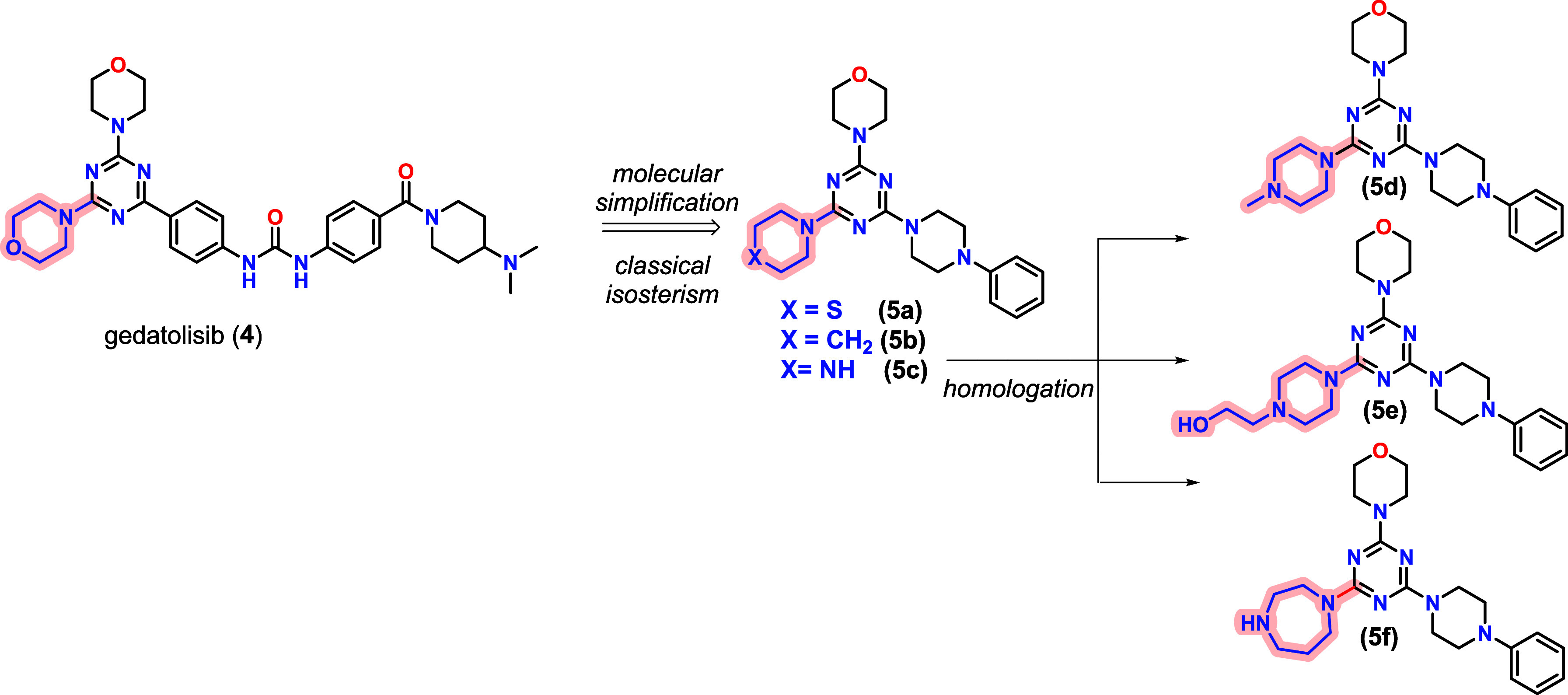
Design concept of compounds **5a**–**f** using gedatolisib (**4**) as a prototype.

Therefore, despite the progress achieved with dual PI3K/mTOR inhibitors, current candidates still suffer from important limitations such as low aqueous solubility, poor metabolic stability, lack of isoform selectivity, and reduced activity in resistant tumor phenotypes. To address these issues, we designed and synthesized a focused set of morpholino-triazine derivatives (**9a**–**f**), obtained by rational modification of the hit compound **5f**, a simplified gedatolisib analog. Our aim was to investigate whether targeted substitutions on the phenyl moiety could (i) enhance molecular interactions in the affinity site of PI3K and mTOR, (ii) modulate isoform selectivity, (iii) improve cytotoxic activity in tumor cell lines carrying PI3K/AKT/mTOR pathway mutations, including multidrug-resistant phenotypes, and (iv) provide preliminary data on permeability and metabolic stability. This integrative approach was intended to generate early SAR and DMPK insights that would be useful for future optimization of dual PI3K/mTOR inhibitors. In this context, we proposed structural modifications on compound **5f**, where the phenylpiperazine subunit was replaced by a phenyl ring containing hydrogen bond donor or acceptor substituents (**9a**), aiming to gain additional interactions with the PI3K affinity site. To further explore the importance of conformational flexibility, the corresponding aza-homologues (**9b**–**f**)^18^ were designed (Figure 3).

**Figure 3:**
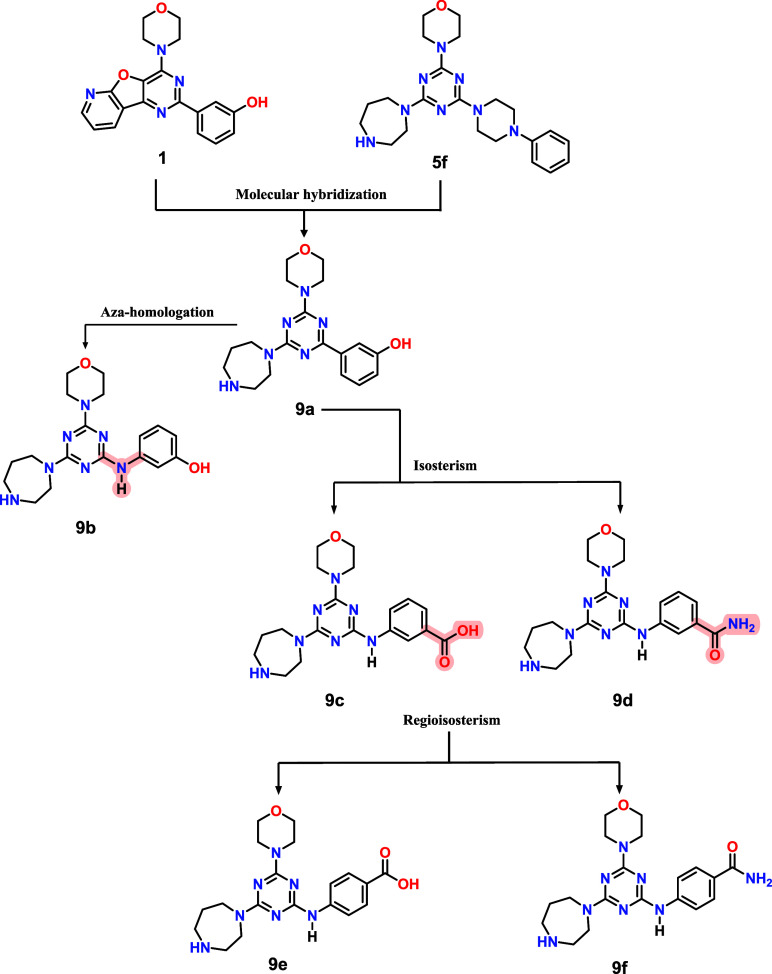
Design concept of compounds **9a**–**f** using **5f** and PI-103 (**1**) as a prototype.

## 2 Experimental Section

### 2.1 Chemistry

All solvents and reagents obtained from commercial sources were used without further purification. Flash column chromatography was performed by using silica (200–300 mesh). ^1^H NMR and ^13^C NMR spectra were recorded on a Bruker AV 500 or Varian 400-MR spectrometer and were calibrated using TMS or residual deuterated solvent as an internal reference (CDCl_3_: ^1^H, δ = 7.26 ppm; DMSO-*d*_6_: δ = 2.50 ppm; acetic acid-*d*_4_: δ = 2.03, 11.53 ppm). The purity of the synthesized compounds was evaluated using a high-performance liquid chromatography (SHIMADZU, LC-20AD 3D) equipped with a Kromasil 100–5C18, and the purity of the biologically tested compounds was ≥95%.

^1^H NMR spectral data are reported in terms of chemical shift (δ, ppm), multiplicity, coupling constant (Hz), and integration. ^13^C NMR spectral data are reported in terms of chemical shift (d, ppm) and multiplicity. Peaks were labeled as singlet (s), doublet (d), triplet (t), quartet (q), and multiplet (m). High-resolution mass spectra were performed on a QExactive Hybrid Quadrupole Orbitrap mass spectrometer.

#### 2.1.1 Preparation of 4-(4,6-Dichloro-1,3,5-triazin-2-yl) Morpholine (**7**)

A solution containing triethylamine (1.0 mL, 7.33 mmol) and morpholine (0.6 mL, 7.4 mmol) in acetonitrile was added dropwise to an acetonitrile solution containing cyanuric chloride (6) (1.5 g, 8.15 mmol). The mixture was stirred in an ice bath for 2 h. After the reaction was completed, the organic solvent was concentrated using a rotary evaporator and distilled water was added to the crude product, followed by vacuum filtration. The obtained material was purified by recrystallization from an acetone/water mixture, yielding the final product (**7**) with an 83% yield. ^1^H NMR (400 MHz, CDCl3) δ 3.81 (t, *J* = 4 Hz, 4H); 3.67 (t, *J* = 4 Hz, 4H). ^13^C NMR (100 MHz, CDCl_3_) δ 170.45, 164.17, 66.32, 44.47.

#### 2.1.2 Preparation of 4-(4-Chloro-6-(1,4-diazepan-1-yl)-1,3,5-triazin-2-yl) Morpholine (**8a**)

To a solution containing 4-(4,6-dichloro-1,3,5-triazin-2-yl) morpholine (**7**) in acetonitrile (15 mL) were added a saturated NaHCO_3_ solution (1:1) and 1 equiv of homopiperazine. The reaction mixture was stirred at room temperature, and upon completion, the organic solvent was concentrated. The solid formed in the aqueous phase was filtered under vacuum, yielding the final product (**8a**) with a 70% yield. ^1^H NMR (400 MHz, CDCl3) δ 3.81 (t, *J* = 4 Hz, 4H); 3.67 (t, *J* = 4 Hz, 4H). ^13^C NMR (100 MHz, CDCl_3_) δ 170.45, 164.17, 66.32, 44.47.

#### 2.1.3 Synthesis of 3-(4-(1,4-Diazepan-1-yl)-6-morpholino-1,3,5-triazin-2-yl) Phenol (**9a**)

To a solution of 4-(4-chloro-6-(1,4-diazepan-1-yl)-1,3,5-triazin-2-yl) morpholine (**8**) (0.5 g; 1.7 mmol) in 50 mL of an acetonitrile/water mixture (1:1) were added Na_2_CO_3_ (0.7 g, 6.8 mmol), 3-hydroxyphenylboronic acid (0.2 g, 1.7 mmol), and the palladium catalyst PdCl_2_(PPh3)_2_ (0.05 g). The reaction mixture was stirred magnetically at 200 °C, and after 4 h, the reaction was observed to be complete. The organic solvent was concentrated, resulting in a precipitate in the aqueous phase which was filtered under vacuum and purified by column chromatography to obtain the final product with a 70% yield. ^1^H NMR (500 MHz, DMSO-*d*_6_) δ 9.95–9.52 (m, 1H), 7.78–7.73 (m, 2H), 7.28–7.16 (m, 1H), 6.94–6.89 (m, 1H). ^13^C (125 MHz, DMSO-*d*_6_) δ 169.58, 164.91, 164.70, 157.43, 138.64, 129.50, 119.15, 118.64, 114.94, 66.17, 46.69, 46.05, 45.62, 45.44, 43.41, 25.67. HRMS (ESI): *m*/*z* [M + H]^+^ calcd. for [C_18_H_24_N_6_O_2_]^+^: 357.1994, found: 357.2029

#### 2.1.4 General Procedure for Obtaining Disubstituted Triazines (**8b-f**)

To a solution containing 1 equiv of 4-(4,6-dichloro-1,3,5-triazin-2-yl) morpholine (**7**) in acetonitrile were added a saturated NaHCO_3_ solution (1:1) and 1.5 equiv of the appropriate aniline. The reaction was stirred at room temperature, and upon completion, the organic solvent was concentrated. The solid formed in the aqueous phase was filtered, purified by recrystallization from hot ethanol, characterized, and used in subsequent steps.

##### 2.1.4.1 3-((4-Chloro-6-morpholino-1,3,5-triazin-2-yl)­amino)­phenol (**8b**)

83% isolated yield, white solid; ^1^H NMR (500 MHz, DMSO-*d*_6_) δ 9.95 (sl, H-1H), 9.48 (s, 1H), 7.16 (s, 1H), 7.08 (t, *J* = 8 Hz, 1H), 7.03–7.01 (m, 1H), 6.45 (dd, *J* = 8 Hz, *J* = 2 Hz, 1H), 3.75–3.50 (m, H-4, 8H); ^13^C NMR (125 MHz, DMSO-*d*_6_) δ 168.48, 164.30, 163.51, 157.60, 139.79, 129.49, 111.22, 110.52, 107.52, 65.93, 43.86.

##### 2.1.4.2 3-((4-Chloro-6-morpholino-1,3,5-triazin-2-yl)­amino)­benzoic acid (**8c**)

75% isolated yield, white solid. ^1^H NMR (500 MHz, DMSO-*d*_6_) δ 9.97 (s, 1H), 8.44 (s, 1H), 7.76 (dd, *J* = 8, *J* = 2 Hz, 1H), 7.62 (d, *J* = 8 Hz, 1H), 7.42 (t, *J* = 8 Hz, 1H), 3.82–3.72 (m, 4H), 3.69–3.64 (m, 4H). ^13^C NMR (125 MHz, DMSO-*d*_6_) δ 172.15, 167.24, 164.11, 139.07, 131.24, 128.94, 124.18, 123.82, 121.10, 65.72, 43.84.

##### 2.1.4.3 3-((4-Chloro-6-morpholino-1,3,5-triazin-2-yl)­amino)­benzamide (**8d**)

85% isolated yield, white solid; ^1^H NMR (500 MHz, DMSO-*d*_6_) δ 10.24 (s, 1H), 8.33 (s, 1H), 7.92 (s, 1H), 7.70 (d, *J* = 8 Hz, 1H ), 7.54 (d, *J* = 8 Hz, 1H), 7.38 (t, *J* = 8 Hz, 1H), 7.33 (s, 1H), 3.79 (s, 1H), 3.72 (s, 2H), 3.69–3.62 (m, 4H); ^13^C NMR (125 MHz, DMSO-*d*_6_) δ 168.55, 167.89, 164.09, 163.52, 138.76, 134.96, 128.45, 122.94, 121.84, 120.11, 65.72, 43.77.

##### 2.1.4.4 4-((4-Chloro-6-morpholino-1,3,5-triazin-2-yl)­amino)­benzoic acid (**8e**)

80% isolated yield, white solid; ^1^H NMR (400 MHz, DMSO-*d*_6_) δ 10.07 (s, 1H), 7.89 (d, 2H), 7.75 (d, 2H), 3.77–3.74 (m, 4H), 3.68–3.66 (m, 4H); ^13^C NMR (100 MHz, DMSO-*d*_6_) δ 168.75, 167.17, 164.22, 163.67, 143.06, 130.45, 124.92, 119.60, 65.90, 43.93.

##### 2.1.4.5 4-((4-Chloro-6-morpholino-1,3,5-triazin-2-yl)­amino)­benzamide (**8f**)

88% isolated yield, white solid; ^1^H NMR (400 MHz, DMSO-*d*_6_) δ 10.29 (s, 1H), 7.87 (s, 1H), 7.84 (d, *J* = 8 Hz, 2H), 7.70 (d, *J* = 8 Hz, 2H), 7.23 (s, 1H), 3.78–3.74 (m, 2H), 3.73–3.69 (m, 2H), 3.69–3.65 (m, 2H), 3.64 (d, *J* = 4 Hz, 2H); ^13^C NMR (100 MHz, DMSO-*d*_6_) δ 168.69, 167.69, 164.23, 163.62, 141.58, 128.55, 128.44, 119.43, 65.90, 65.80, 44.00, 43.87.

#### 2.1.5 General Procedure for Obtaining the Final Compounds (**9b**–f)

To a mixture containing 1 equiv of the disubstituted intermediate (**8b**–**f**) in dioxane/water (1:1), 1.5 equiv of homopiperazine and 2.5 equiv of Na_2_CO_3_ were added. The resulting mixture was stirred and refluxed, and the reaction was observed to be complete after 6 h. The solution was concentrated under a vacuum, and the crude product was purified by column chromatography on silica gel (dichloromethane/methanol = 10:1).

##### 2.1.5.1 3-((4-(1,4-Diazepan-1-yl)-6-morpholino-1,3,5-triazin-2-yl)­amino)­phenol (**9b**)

60% isolated yield, white solid; HPLC purity: 99.2%; M.p.: 215–217 °C; ^1^H NMR (500 MHz, DMSO-*d*_6_) δ 11.02 (s, 1H), 10.67 (s, 1H), 7.18 (t, *J* = 8 Hz, 1H), 7.00 (t, *J* = 2.2 Hz, 1H), 6.91 (ddd, *J* = 8, *J* = 2 Hz, 1H), 6.63 (ddd, *J* = 8, *J* = 2 Hz, 1H), 3.80–3.61 (m, 16H), 3.11–3.06 (m, 2H); ^13^C NMR (125 MHz, DMSO-*d*_6_) δ 164.72, 164.44, 164.00, 157.35, 141.57, 128.87, 110.36, 108.58, 106.61, 66.01, 65.87, 62.05, 47.21, 46.47, 43.32, 25.50; HRMS (ESI): *m*/*z* [M + H]^+^ calcd. for [C_18_H_25_N_7_O_2_]^+^: 372.2103, found: 372.2147.

##### 2.1.5.2 3-((4-(1,4-Diazepan-1-yl)-6-morpholino-1,3,5-triazin-2-yl)­amino)­benzoic acid (**9c**)

50% isolated yield, white solid; HPLC purity: 99.7%; M.p.: 250–252 °C; ^1^H NMR (500 MHz, DMSO-*d*_6_) δ 9.43 (1H, s, H-22), 8.95 (s, 1H), 8.58 (s, 1H), 7.67 (d, *J* = 8 Hz, 1H), 7.55 (d, *J* = 8 Hz, 1H), 7.38 (t, *J* = 8 Hz, 1H), 4.02–3.95 (m, 2H), 3.87–3.80 (m, 2H), 3.78–3.68 (m, 4H), 3.68–3.60 (m, 4H), 3.27 (s, 2H), 3.18 (s, 2H), 2.09–2.04 (m, 2H); ^13^C NMR (125 MHz, DMSO-*d*_6_) δ 167.40, 167.29, 163.24, 162.10, 139.78, 131.21, 128.72, 123.76, 123.03, 120.75, 65.94, 45.14, 44.82, 44.72, 43.80, 42.75, 24.84; HRMS (ESI): *m*/*z* [M + H]^+^ calcd. for [C_19_H_25_N_7_O_3_]^+^: 400.2052, found: 400.2083.

##### 2.1.5.3 3-((4-(1,4-Diazepan-1-yl)-6-morpholino-1,3,5-triazin-2-yl)­amino)­benzamide (**9d**)

65% isolated yield, white solid; HPLC purity: 98.6%; M.p.: 280–282 °C; ^1^H NMR (400 MHz, DMSO-*d*_6_) δ 8.85 (s, 1H), 8.38 (s, 1H), 7.71 (d, *J* = 8 Hz, 1H), 7.41 (d, *J* = 8 Hz, 1H), 7.28 (t, *J* = 8 Hz, 3H), 4.10–3.40 (m, 16H), 1.94 (q, *J* = 11, *J* = 8 Hz, 2H); ^13^C NMR (100 MHz, DMSO-*d*_6_) δ 168.46, 164.73, 164.52, 164.07, 140.58, 134.87, 128.07, 121.97, 120.15, 119.07, 66.03, 47.33, 46.15, 45.77, 45.22, 43.32, 30.74; HRMS (ESI): *m*/*z* [M + H]^+^ calcd. for [C_19_H_26_N_8_O_2_]^+^: 399.2212, found: 399.2241.

##### 2.1.5.4 4-((4-(1,4-Diazepan-1-yl)-6-morpholino-1,3,5-triazin-2-yl)­amino)­benzoic acid (**9e**)

60% isolated yield, white solid; HPLC purity: 98.4%; M.p.: 218–220 °C; ^1^H NMR (500 MHz, Ácido acético-*d*_4_) δ 8.04 (d, *J* = 8 Hz, 2H), 7.79 (d, *J* = 8 Hz, 2H), 4.13 (t, *J* = 5 Hz, 2H), 3.95 (t, *J* = 6 Hz, 2H), 3.87–3.78 (m, H-6, 8H), 3.55 (t, *J* = 5 Hz, 2H), 3.43 (t, *J* = 5 Hz, 2H), 2.28–2.23 (m, 2H); ^13^C NMR (125 MHz, Ácido acético-*d*_4_) δ 170.54, 163.96, 163.57, 163.57,144.29, 130.96, 123.25 (C-20), 119.25, 66.19, 46.05, 45.62, 45.00, 44.02, 42.90, 24.92; HRMS (ESI): *m*/*z* [M + H]^+^ calcd. for [C_19_H_25_N_7_O_3_]^+^: 400.2052, found: 400.2089.

##### 2.1.5.5 4-((4-(1,4-Diazepan-1-yl)-6-morpholino-1,3,5-triazin-2-yl)­amino)­benzamide (**9f**)

70% isolated yield, white solid; HPLC purity: 98.5%; M.p.: 300 °C; ^1^H NMR (400 MHz, Ácido acético-*d*_4_) δ 7.89 (d, *J* = 8 Hz,2H), 7.76 (d, *J* = 8 Hz, 2H), 4.13 (t, *J* = 5 Hz, 2H), 3.96 (t, *J* = 6 Hz, 2H), 3.88–3.82 (m, 4H), 3.80 (d, *J* = 5 Hz, 4H), 3.55 (t, *J* = 5 Hz, 2H), 3.43 (t, *J* = 5 Hz, 2H), 2.28–2.22 (m, 2H), 1.33 (sl, 1H); ^13^C NMR (100 MHz, DMSO-*d*_6_) δ 167.60, 164.68, 164.45, 163.92, 143.33, 128.12, 126.69, 118.16, 65.99, 46.49, 46.22, 45.76, 45.08, 43.36, 25.72; HRMS (ESI): *m*/*z* [M + H]^+^ calcd. for [C_19_H_26_N_8_O_2_]^+^: 399.2212, found: 399.2249.

### 2.2 Kinetic Solubility

The kinetic solubility of the compounds investigated in this study was assessed through the incubation of a 200 μM stock solution containing 10 mM of each compound in a 1 mL reaction volume, prepared using a 0.1 M phosphate buffer at pH 7.4. Incubation periods of 4 and 24 h were employed under constant agitation at room temperature.^19^

Following this, six dilutions were generated from the 200 μM solution to construct a calibration curve and to facilitate the linear regression analysis. After the incubation and preparation of the diluted solutions, all samples were filtered by using a 0.23 μm filter and subsequently analyzed by using high-performance liquid chromatography (HPLC). The solubility of the compounds was determined based on the linear regression equation derived from the calibration curve.^19,20^

### 2.3 Biological Evaluation

#### 2.3.1 PI3K/mTOR Inhibition Biochemical Assays

The HotSpot Kinase Assay was used for compound screening on protein kinases, as already reported.^21^ The assay was performed in a base reaction buffer containing 20 mM Hepes (pH 7.5), 10 mM MgCl2, 1 mM EGTA, 0.01% Brij35, 0.02 mg/mL BSA, 0.1 mM Na_3_VO_4_, 2 mM DTT, and 1% DMSO, with the required cofactors added individually to each kinase reaction. The substrate was prepared in a freshly made base reaction buffer, and cofactors were added accordingly. The indicated kinase was then delivered to the substrate solution and gently mixed. Test compounds, dissolved in 100% DMSO, were added to the kinase reaction mixture using acoustic technology (Echo550; nanoliter range), followed by incubation for 20 min at room temperature. The reaction was initiated by adding 33P-ATP (1 μM) and incubating it for 2 h at room temperature. Kinase activity was detected by using the P81 filter-binding method.

The phosphoinositide 3-kinase (PI3K) activity was measured by using the ADP-Glo Kinase Assay (Promega). Reactions were set up in a 96-well white plate with a final volume of 25 μL per well, containing kinase reaction buffer (40 mM Tris-HCl, pH 7.5, 20 mM MgCl_2_, 0.1 mg/mL BSA, 0.01% Tween-20, 1 mM EGTA, and 50 μM DTT), 100 μM ATP, 20 μM phosphatidylinositol-4,5-bisphosphate (PIP_2_) as substrate, recombinant PI3K enzyme, and test inhibitors or DMSO (vehicle control). After all components were added, the reactions were incubated at room temperature for 60 min. The reactions were terminated by adding 25 μL of ADP-Glo Reagent to deplete unreacted ATP, followed by a 40 min incubation. Then, 50 μL of a Kinase Detection Reagent was added, converting ADP to ATP and producing a luminescent signal via luciferase. After 30 min, luminescence was measured using a microplate reader. Raw luminescence values were normalized to controls (no enzyme and no inhibitor), and inhibition curves were generated to determine IC_50_ values.

All compounds were initially tested at a single concentration (10 μM), using the pan dual PI3K and mTOR inhibitor PI-103 (**1**) as a positive control.^22^ The compounds that showed ≥50% inhibition of PI3K and/or mTOR were selected for determination of the concentration–response curve and calculation of the IC_50_.

#### 2.3.2 Cell Lines and Cell Cultures

The tumor cell lines used in this study were acute lymphoblastic leukemia with a mutation in PTEN (CCRF-CEM), acute lymphoblastic leukemia with mutations in PTEN and PIK3R1 (MOLT-4), breast cancer with a mutation in PIK3CA (MCF-7), prostate cancer with a mutation in PTEN (PC-3), and chronic myelogenous leukemia (CML) cell line K562 with mutations in P53/CDKN2A. In all experiments, cells were cultured in Roswell Park Memorial Institute (RPMI 1640 medium) (Gibco, MA, USA, cat. 11875), supplemented with 10% fetal bovine serum (FBS). This medium is called the proper medium of this cell, with 1% penicillin antibiotic (10 U/mL) and streptomycin (10 μg/mL) (Gibco, MA, USA, cat. 15140122). The chronic myelogenous leukemia cell line Lucena (K562-Lucena or K562/VIN) was established from the K562 cell line, under the pressure of vincristine supplement in the culture medium. It expresses the P-glycoprotein and has a multidrug-resistance (MDR) phenotype. For subcultures, (2–5) × 10^4^ cells/mL were harvested every 3 days, kept at 37 °C with a humid atmosphere, containing 5% CO_2._ The cells were maintained in the proper medium and supplemented with 60 nM of vincristine. The chemotherapeutic drug vincristine (VIN) (cat. no. V8388) was purchased from Sigma-Aldrich (St. Louis, MO, USA). For the renewal of the culture medium, a subculture ratio of 1:4 was used, every 3 days in 75 cm^2^ flasks, and maintained at 37 °C, with a humid atmosphere containing 5% CO_2_. All experiments were conducted with biosafety level 1. Immortalized cells were also obtained from the Rio de Janeiro Cell Bank (BCRJ).

#### 2.3.3 Cell Viability Assay by MTT Assay

The 3-(4,5-dimethylthiazol-2-yl)-2,5-diphenyl-tetrazolium bromide (MTT) assay was performed as previously described and modified^23^ in order to evaluate the cellular metabolic activity in the presence of the compounds of this study that were added at different concentrations ranging from 0.003 μM to 100 μM. This colorimetric method is based on the principle of transforming the yellow salt of tetrazolium MTT into purple formazan crystals, due to the metabolic activity of living cells by pyridine nucleotide cofactors NADH and NADPH and mitochondrial dehydrogenase. The formazan crystals formed were dissolved in a detergent solution, and the absorption of the color solution was measured quantitatively using a plate reader at 595 nm. Thus, in this work, after treatment of the cells with the compounds, the cells were centrifuged at 440 × g for 10 min at 4 °C (Universal centrifuge 320R Hettich, Kirchlengern, Germany), and 110 μL of supernatant were carefully removed and 10 μL MTT reagent (5 mg/mL in PBS) were added to each well. Then, the system was protected from light and kept for 3.5 h in a CO_2_ atmosphere at 37 °C. After incubation, 100 μL of detergent solution (SDS/HCl 0.1 g/mL) were added to solubilize the produced formazan crystals. The plate was protected from light at room temperature for 24 h. Finally, the light absorption of each well was evaluated with a plate reader (Molecular Devices Spectramax M5 plate reader). MTT reagent (catalog number M5655) was purchased from Sigma-Aldrich (St. Louis, MO, USA). All procedures performed with CCRF-CEM, PC-3, MOLT-4, K562, Lucena, and MCF7 cells were conducted in vitro.

#### 2.3.4 Preparation of Compound Solutions

For the assays, a stock solution of 10 mM of the compounds, except gedatolisib that was at 5 mM, was prepared in 100% dimethyl sulfoxide (DMSO). From that stock, five other solutions in 100% DMSO were prepared, at concentrations of 3, 0.3, 0.03, 0.003, and 0.0003 mM of the compound. These solutions were diluted in a proper medium to obtain per well 1% DMSO at final concentrations of 100, 30, 3, 0.3, 0.03, and 0.003 μM. A concentration of 1% or less is not toxic to human cell line culture.^24^ The DMSO reagent (cat. no. 472301) was purchased from Sigma-Aldrich (St. Louis, MO, USA).

#### 2.3.5 Procedure for Isolating Human Monocytes from Peripheral Blood (hPBMC)

In this study, peripheral blood mononuclear cells (hPBMCs) were used as a nontumor cell model. These cells are easily isolated by density gradient centrifugation.^25^ The hPBMC blood is diluted, treated with anticoagulant, and layered in Ficoll-Paque PLUS solution (GE Healthcare, IL, USA) and centrifuged. During centrifugation, erythrocytes and granulocytes sediment to the lower layer. Lower-density lymphocytes, along with other slow sedimenting cells such as platelets and monocytes, are retained at the interface between the plasma and the Ficoll-Paque, where they can be collected. For this assay, about 20 mL of blood from the donors were collected and carefully loaded into a 50 mL plastic tube containing 25 mL of Ficoll-Paque Plus and centrifuged at 900 × g for 30 min at 20 °C. Finally, the separated mononuclear fraction was collected and diluted with PBS, containing 3 mM EDTA, followed by centrifugation at 500 × g for 10 min. After that, the sample was analyzed by MTT assay, according to Section 2.3.3. Human peripheral blood mononuclear cells (PBMCs) were obtained according to a protocol approved by the Ethics Committee CAAE 48 917 418 2 0000 5257.

#### 2.3.6 Statistical Analysis

Statistical analyses were performed using GraphPad Prism (Software version 9, San Diego, CA, USA). Each experiment was tested in triplicate in three independent experiments (N3). Data were represented as mean ± SD, confidence interval, and analyzed using descriptive statistics and nonlinear regression (log inhibitor vs response) to compare the differences. Values of *p* ≤ 0.05 were accepted as statistically significant.

### 2.4 Docking Analysis

For the docking analysis, the crystal structure of PI3Kα (PDB ID: 4L23) and the crystal structure of mTOR (PDB ID: 4JT6) were selected. From the cocrystallized ligands, a 10 Å radius was used for binding site selection. A redocking analysis of the cocrystallized ligands for each crystal was performed to select the best scoring function available on GOLD’2022.3.0 based on RMSD values. The selected scoring function was GoldScore for PI3Kα and mTOR.

### 2.5 Parallel Artificial Membrane Permeability Assay (PAMPA)

Compounds’ permeability profile through the gastrointestinal tract (GIT) and blood–brain barrier (BBB) was assessed using the parallel artificial membrane permeability assay.^26,27^

#### 2.5.1 PAMPA BBB

One mg portion of each compound (test or control) was dissolved in 1 mL of ethanol. Then, 500 μL of ethanol and 3.5 mL of PBS at pH 7.4 were added. The solution was then filtered (PVDF filter: 0.45 μM) and set aside. Subsequently, 180 μL of a solution of PBS pH 7.4: ethanol (70:30) was added to the wells of the recipient plate, and 5 μL of the pig brain lipid solution (20 mg lipid/mL, in dodecane) was added to the wells of the donor plate. After 5 min, the donor plate received, in triplicate, 180 μL of the solution containing each compound. The donor plate was then carefully placed on top of the recipient plate, forming a sandwich system, which was left to stand for 2 h and 45 min at room temperature (±25 °C) in a closed container containing 10 mL of PBS pH 7.4. After this period, the donor plate was removed, and the contents of the recipient plate were transferred to a UV reading plate (SpectraMax 5, Molecular Devices), which was read at the wavelengths previously established for each compound. The blank was prepared in the presence of 180 μL of PBS (pH 7.4): ethanol (70:30) solution (adapted from Di et al., 2003). The PAMPA-BBB model only classifies compounds as permeable (BBB+) or nonpermeable (BBB−).^27,28^

#### 2.5.2 PAMPA GIT

250 μL portion of the solution of each compound (test or control) at 10 mM in DMSO was homogenized with 4750 μL of PBS, pH 6.6, at 10 mM. The solution was then filtered (PVDF filter: 0.45 μM) and set aside. Subsequently, 180 μL of a solution of PBS, pH 7.4: DMSO (95:5), was added to the wells of the recipient plate, and 5 μL of the solution of the lipid l-α-phosphatidylcholine from soy (20 mg lipid/mL, in dodecane) was added to the wells of the donor plate. After 5 min, the donor plate received, in triplicate, 180 μL of the reserved solution containing each compound. The donor plate was then carefully placed on top of the recipient plate, forming a “sandwich” system, which was left to shake at 50 rpm for 8 h at room temperature (±25 °C), in a closed container containing 10 mL of PBS, pH 7.4. After this period, the donor plate was removed and the contents of the receiver plate were transferred to a UV reading plate (SpectraMax 5, Molecular Devices), which was read at the wavelengths previously established for each compound. The blank was prepared in the presence of 180 μL of PBS solution (pH 7.4): DMSO (95:5) (adapted from Sugano et al., 2001; Fortuna et al., 2011). The permeability result for PAMPA-TGI classifies the compounds according to the percentage of fraction absorbed (Fa%) as high intestinal permeability (70–100%), medium permeability (30–69%), or low permeability (0–29%).^29^

#### 2.5.3 Data Analysis

The optical density values obtained in the reading at each selected wavelength for each of the compounds were analyzed in comparison with the values of the controls. In the case of BHE, these values were used to draw up a straight-line equation and determine the permeability coefficient (Pe), while for TGI, these values were used to determine the absorbed fraction (Fa %), both assays using a previously prepared Excel spreadsheet. The experiments were carried out in triplicate and in two different analyses (*n* = 2) in the presence of controls.^27,30^

### 2.6 Microsomal Stability

Microsomal stability was evaluated by incubating 10 μM of a 1 mM stock solution of **9a** (LASSBio-2337) and **9b** (LASSBio-2338) with rat liver microsomes (protein concentration: 1 mg/mL) in the presence of an NADPH-regenerating system. The reaction mixture contained 1.3 mM MgCl2, 0.4 mM NADP+, 3.5 mM glucose-6-phosphate, and 0.5 U/mL glucose-6-phosphate dehydrogenase, all prepared in 0.1 M phosphate buffer (pH 7.4). The total reaction volume was adjusted to 250 μL.^32,33^

The experimental protocol involved an initial preincubation of the samples at 37 °C, followed by incubation at the same temperature under continuous agitation for predetermined time intervals (0, 15, 30, 45, and 60 min). The enzymatic reactions were terminated by the addition of 1 mL of a solution composed of acetonitrile and methanol (1:1, v/v) containing 2 μM internal standard. This step ensured both the extraction of analytes and the precipitation of proteins. The mixtures were subsequently subjected to centrifugation at 24,500 × g for 15 min at 4 °C to achieve phase separation. The supernatant (1 mL) was carefully collected, filtered, and analyzed by high-performance liquid chromatography (HPLC). The procedure was performed both in the presence and absence of enzymatic cofactors and conducted in triplicate to ensure reproducibility.^31,32^ Rat liver microsomes were obtained according to a protocol approved by the Ethics Committee on the Use of Animals in Research at the Federal University of Rio de Janeiro, CEUA-UFRJ 046/21.

## 3 Results and Discussion

### 3.1 Synthetic Chemistry

The synthetic route for obtaining the target compounds is outlined in Scheme 1. Synthesis of the morpholino-triazine compounds **9a**–**f** was achieved by the general synthetic route involving a combination of Suzuki coupling and nucleophilic substitution on the commercially available cyanuric chloride **6**. Initially, two chlorines in cyanuric chloride **6** were replaced by morpholine and homopiperazine, which allowed mono- and disubstituted intermediates **7** and **8a**, respectively. The third chloride in compound **8a** was displaced using the Suzuki coupling reaction with 3-hydroxyphenylboronic acid to yield **9a.**

**Scheme 1:**
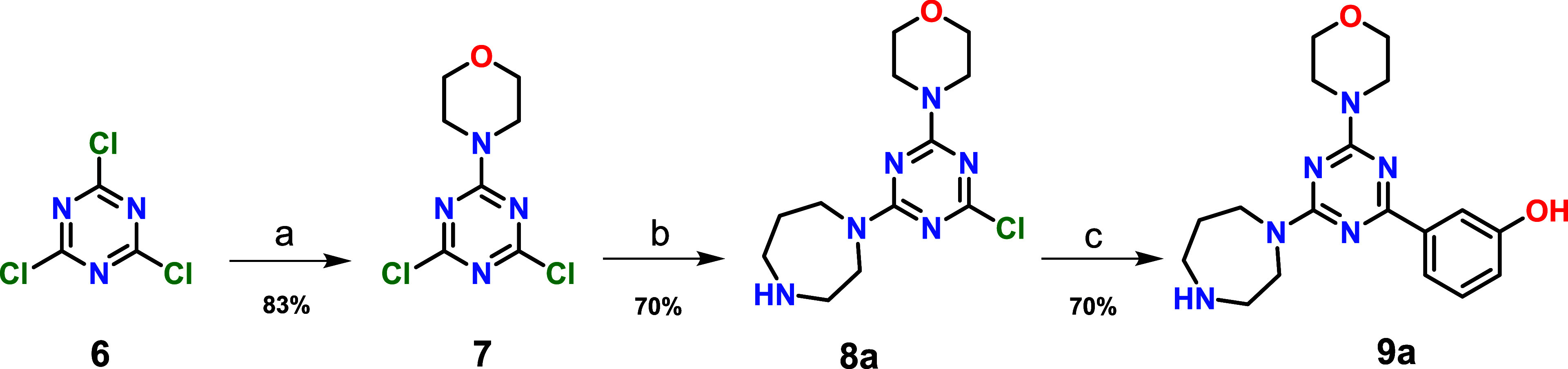
General Methodology for Synthesis of Compound **9a** ^a^ ^b^ ^c^

a Morpholine, Et_3_N, acetonitrile-H_2_O (1:1), 0 °C, 1 h;
b homopiperazine, acetonitrile/NaHCO_3_ (aq) (1:1), rt, 4 h;
c 3-hydroxyphenylboronic acid, PdCl_2_(PPh_3_), Na_2_CO_3_, acetonitrile-H_2_O (1:1), 100 °C.

Alternatively, intermediate **7** was reacted with different amines to obtain intermediates **8b**–**f**. These intermediates were subjected to substitution of the third chlorine with homopiperazine to obtain the final compounds **9b**–**f** (Scheme 2). These final products were purified by recrystallization or by flash column gel chromatography. **9a**–**f** were analyzed for purity by analytical HPLC on a Kromasil column 100–5C18 (250 mm × 4.6 mm) using an acetonitrile/methanol mixture.

**Scheme 2:**
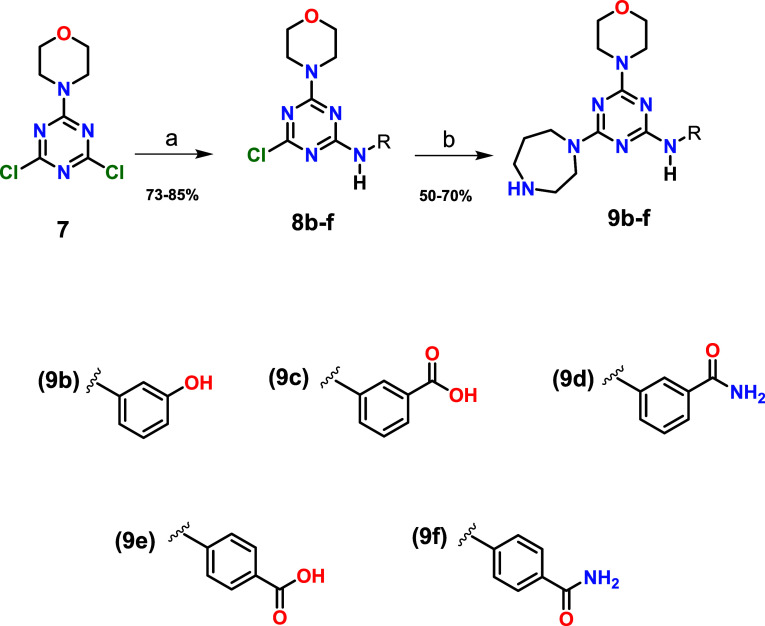
General Methodology for Synthesis of Compounds **9b**–**f** ^a^ ^b^

a Aniline, acetonitrile/NaHCO_3_ (aq) (1:1), rt, 8 h;
b homopiperazine, Na_2_CO_3_, dioxane-H_2_O (1:1), 100 °C, 4 h.

### 3.2 Kinetic Solubility

Compound **9a** and its aza-homologue (**9b**) were insoluble at pH 7.4, exhibiting aqueous solubility lower than 1 μM (Table 1). The replacement of the hydroxyl group at the *meta*-position of the phenyl ring (**9a**) by a carboxylic acid (**9c**) or by an amide resulted in a significant increase in aqueous solubility. The increase of more than 80 times found for **9c** may suggest that at pH 7.4, the carboxylic acid group is ionized, favoring solubility. The amide group (nonionized) in **9d** contributed to an increase in solubility of around 9 times. Both substituents, when positioned in *para*, result in increased aqueous solubility. When we analyzed the solubilization dynamics (at 4 h and 24 h), we found that solubility changed over time, leading to the precipitation of **9d** and **9f**.

**Table 1:** Kinetic Solubility of Compounds **9a**–**9f** Determined at 4 h and 24 h

	Solubility pH 7.4 (μM)			
Compounds	4 h	24 h	cLogP^a^	p*K*_a_ (carboxylic acid)^a^
9a	<1	<1	0.95	
9b	<1	<1	0.54	
9c	82.83	76.95	0.87	3.9
9d	9.52	1.06	0.22	
9e	51.47	51.93	0.87	3.9
9f	14.16	<1	0.22	

a Calculated using the ACD/Percepta 14.0.0 program.

### 3.3 Biological Evaluation

#### 3.3.1 PI3K/mTOR Inhibition Study

The inhibitory activity of the target compounds against all isoforms of PI3K and mTOR was investigated. For PI3K inhibition activity, the ADP-Glo luminescent kinase assay was used. While for mTOR inhibition activity, the HotSpot methodology was employed. All compounds were initially tested at a single concentration (10 μM), using the pan dual PI3K and mTOR inhibitor PI-103 (**1**) as a positive control.^22^ The compounds that showed ≥50% of inhibition of PI3K and/or mTOR were selected for the determination of the concentration–response curve and calculation of the IC_50_. The inhibitory activity of hit **5f** was also evaluated, since its ability to inhibit PI3K and/or mTOR in an enzymatic model had not been investigated previously.

In agreement with the phenotypic studies published by Marques et al.,^17^ the enzymatic inhibition data obtained for the hit **5f** revealed its inactivity against the different PI3K isoforms and against mTOR, when tested at a concentration of 10 μM. It is important to note that the ability of **5f** to modulate the phosphorylation step mediated by PI3K in hematological neoplasia cell lines (MOLT-4 and CCRF-CEM) was detected at a concentration of 50 μM, which is five times higher than the one used in the enzymatic screening.^17^

The structural modifications introduced in **5f** resulted in four compounds with an optimized inhibitory profile against PI3K and mTOR, exhibiting IC_50_ values at low micromolar range (Table 2). Compounds **9a** and its aza-homologue **9b** showed good inhibitory activity against all PI3K isoforms, with equipotent inhibition values observed for the δ and γ isoforms. Comparison between **9a** and **9b** reveals that aza-homologation resulted in a slight decrease in inhibitory potency on PI3Kα and PI3Kβ isoforms, and a loss of activity on mTOR. The compound containing the carboxylic acid group in *para* as substituent (**9e**) was inactive on the different PI3K isoforms but exhibited a potency of 9.7 μM on mTOR (Table 2). These data suggest that the presence of ionizable acid substituents is detrimental to the inhibitory effect on PI3K. Conversely, replacing the phenolic hydroxyl group in **9b** with an amide moiety resulted in a more selective inhibitory profile toward the PI3Kα isoform (Table 2). This compound proved to be equipotent in inhibiting PI3Kα and mTOR. When compared with the reference dual inhibitor PI-103 (**1**), which exhibits nanomolar inhibitory potency against all PI3K isoforms and mTOR (IC_50_ = 0.1–8 nM),^22^ the new morpholino-triazine derivatives **9a** and **9b** showed lower inhibition in the low-micromolar range. Nevertheless, both compounds maintained a dual inhibition profile toward PI3K and mTOR, similarly to PI-103 (**1**), and displayed balanced activity across the PI3Kδ and PI3Kγ isoforms, which are functionally relevant in hematological malignancies. Structurally, the simplified scaffold designed from PI-103 (**1**) involved the replacement of the pyridyl-morpholine moiety by a phenyl or aza-phenyl substituent, which preserved the morpholine–Val851 hinge interaction but reduced the number of hydrogen-bond and hydrophobic interactions within the ATP-binding pocket. This structural simplification likely accounts for the decrease in potency relative to that of PI-103 (**1**). Despite this reduction, the simplified triazine derivatives retain the essential pharmacophoric elements of PI-103 (**1**) responsible for dual PI3K/mTOR inhibition while offering advantages in synthetic accessibility and structural flexibility. Therefore, compounds **9a** and **9b** can be considered valuable leads for further optimization aimed at improving potency and pharmacokinetic properties, while maintaining balanced activity against both kinase targets.

**Table 2:**
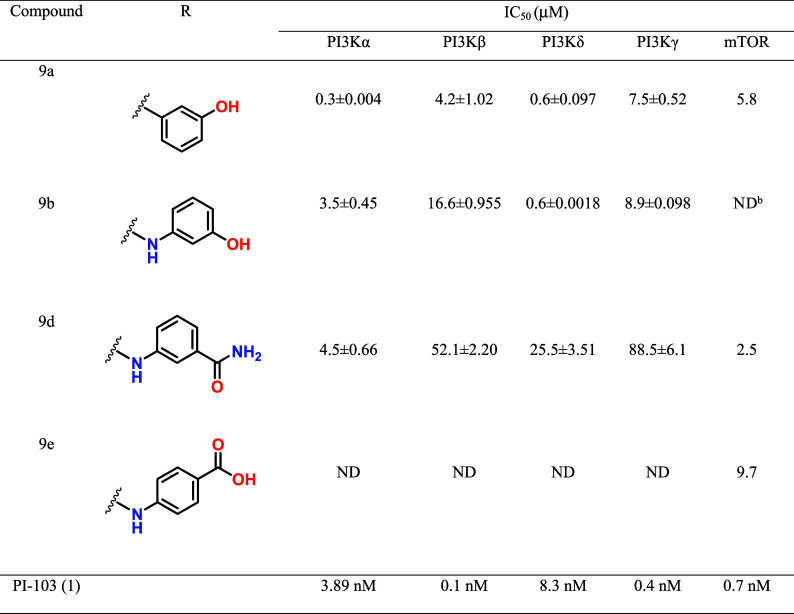
Inhibitory Potency (IC_50_)­^a^ on PI3K Isoforms and on mToR^b^

a IC_50_ values are the mean ± SD of duplicate measurements.

b ND means not determined.

#### 3.3.2 Cell Viability Assay by MTT and CC_50_ Determination

Compounds were screened at 30 μM using cell viability analysis by the MTT assay at 72 h against four human cell lines that had a mutation in the PI3K pathway. Two were hematological neoplasms of the acute lymphoblastic leukemia type (CCRF-CEM with a mutation in PTEN, and MOLT-4 with mutations in PTEN and *PIK3R1*), and the remaining two were breast carcinoma (MCF7 with a mutation in *PIK3CA*) and prostate adenocarcinoma (PC3 with a mutation in PTEN). Gedatolisib (**4**) was used as a standard PI3K and mTOR inhibitor, and 1% DMSO (dimethyl sulfoxide) was used as a vehicle. The results were standardized against DMSO and analyzed using GraphPad Prism 9.0 software and are shown in Figure 4.

**Figure 4:**
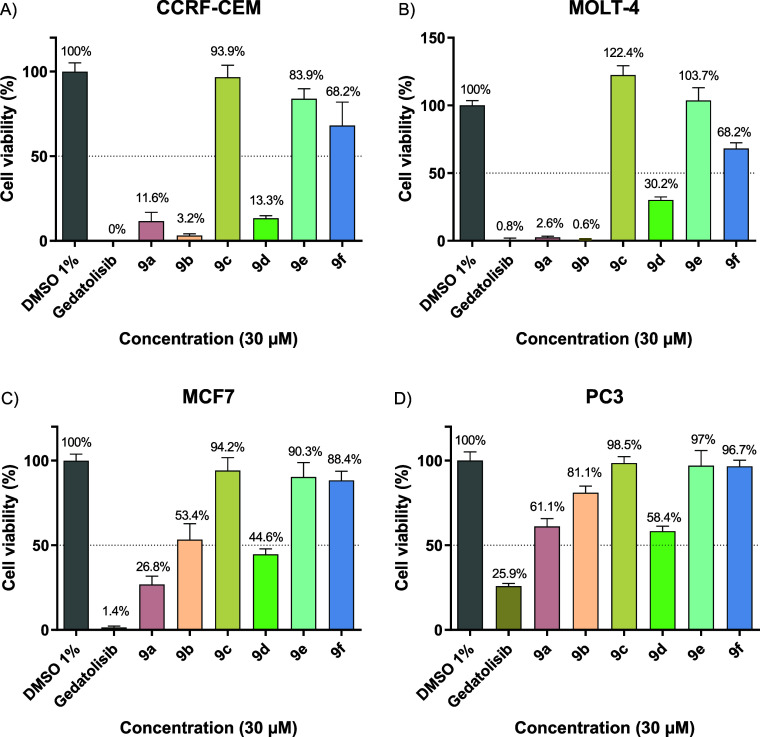
Cell viability analysis by MTT assay at 72 h of the target compounds and standard at a concentration of 30 μM against the strains: (A) CCRF-CEM, (B) MOLT-4, (C) MCF7, and (D) PC3. Data presented as mean ± standard error of the mean of three independent experiments. *p* ≤ 0.05.

The aim of the screening was to select compounds with inhibition of cell viability or cytotoxic effect ≥50% at 30 μM, for subsequent concentration–response assays. As a control, the dual pan-inhibitor gedatolisib (**4**) (IC_50_, PI3Kα = 0.4 nM, IC_50_, PI3Kβ = 6 nM, IC_50_, PI3Kδ = 6 nM, IC_50_, PI3Kγ = 5.4 nM, and IC_50_, mTOR = 1.6 nM^32^) reduced cell viability by ≥70% in the four strains evaluated (Figure 4).

The results for the CCRF-CEM and MOLT-4 human hematological cell lines (Figure 4A,B) indicate greater sensitivity to the target compounds compared with the MCF7 and PC3 solid tumor cell lines (Figure 4C,D), as evidenced by higher percentages of inhibition of cell viability. In the MOLT-4 strain (Figure 4B) with mutations in PTEN and *PIK3R1* (corresponding to the regulatory subunit of PI3Kα), compounds **9a** and **9b** showed comparable inhibition to gedatolisib (**4**), and **9d** displayed inhibition of around 70%. Similar results were observed against CCRF-CEM cells which have a PTEN mutation (Figure 4A). Compounds **9a** (88.4%), **9b** (96.8%), and **9d** (86.7%) showed inhibition of cell viability ≥80%, while gedatolisib (**4**) inhibited 100%.

Compounds **9c** and **9e**, which did not reduce the viability of MOLT-4 cells and were inactive in CCRF-CEM lines, share the presence of a carboxylic acid as a substituent in the *meta* and *para* positions, respectively. The presence of this substituent (p*K*_a_ = 3.9) can impair permeation through cell membranes, since in culture medium (pH = 7), it will be predominantly ionized, decreasing compound’s lipophilicity. In addition, it seems to contribute to the loss of potency over PI3K (Table 2). Compound **9f** (in contrast to its regioisomer **9d**) showed low cytotoxic activity in MOLT-4 and CCRF-CEM cells, inhibiting cell viability by only 30%.

None of the compounds inhibited cell viability by ≥50% on PC3 cells (Figure 4D), while on MCF7 cells (Figure 4C), compounds **9a** and **9d** exceeded 50% inhibition and **9b** reached 46.6%. This activity in CCRF-CEM, MOLT-4, and MCF7 correlates with the PI3Kα inhibition profile (Table 2) of **9a** (IC_50_ = 0.3 μM), **9b** (IC_50_ = 3.5 μM), and **9d** (IC_50_ = 4.5 μM). Compounds **9c**, **9e**, and **9f** were inactive in both solid tumor cell lines.

##### 3.3.2.1 Determination of CC_50_ Using the 72 h MTT Test

Based on the results of the single-concentration screening, compounds **9a**, **9b**, and **9d** were selected to determine their cytotoxic potency in the selected human tumor cell lines. The mean cytotoxic concentration (CC_50_) was determined using the MTT assay after 72 h of incubation. The results are depicted in Table 3 and Figure 5 and were compared with those of the standard gedatolisib (**4**) and with the values previously reported for the hit compound **5f**.^17^

**Figure 5:**
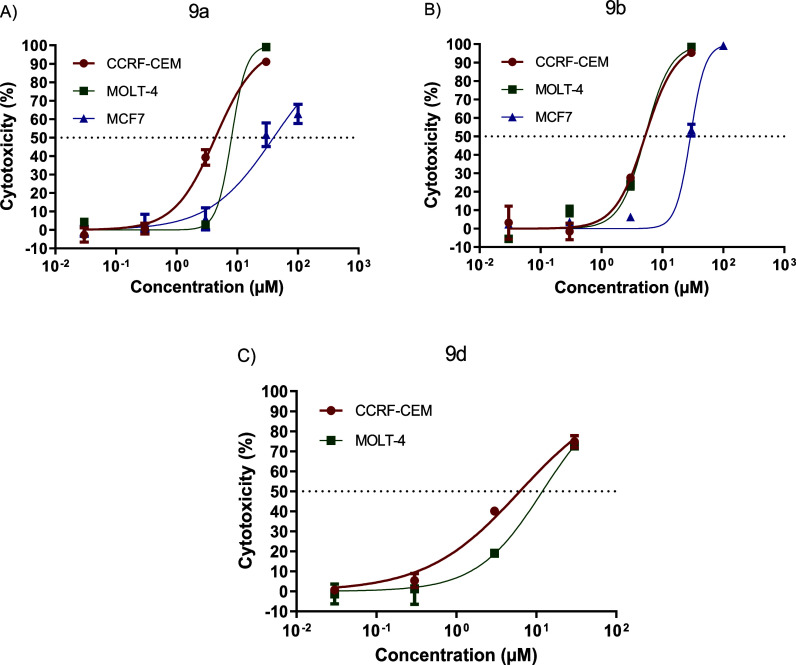
Concentration versus cytotoxicity curve as a function of the variation in concentration of compounds (A) **9a**, (B) **9b**, and (C) **9d**, against the CCRF-CEM, MOLT-4, and MCF7 strains, from the MTT assay over 72 h. Data presented as mean ± standard error of the mean of three independent experiments.

**Table 3:** Cytotoxic Concentration (CC_50_) of **9a**, **9b**, **9d**, and Gedatolisib, against CCRF-CEM, MOLT-4, and MCF-7 Cells, from 72 h Cell Viability Tests and CC_50_ Values Found in the Literature for **5f** ^a^

Compound Cell line	**5f** [18] CC_50_ (μM)	Gedatolisib CC_50_ (μM)	**9a** CC_50_ (μM)	**9b** CC_50_ (μM)	**9d** CC_50_ (μM)
CCRF-CEM	6.25	0.09 (0.08–0.11)	4.37 (3.45–5.53)	5.22 (3.25–8.36)	6.24 (4.54–8.58)
		*E*_max_ = 100%	*E*_max_ = 91.1%	*E*_max_ = 95.3%	*E*_max_ = 74.8%
MOLT-4	9.76	0.07 (0.06–0.08)	8.05 (3.80–17.06)	5.20 (3.29–8.23)	11.91 (7.97–17.79)
		*E*_max_ = 99.8%	*E*_max_ = 99.1%	*E*_max_ = 98.5%	*E*_max_ = 72.6%
MCF7	37.04	0.06 (0.04–0.08)	39.69 (24.72–63.71)	28.89 (26.73–31.22)	
		*E*_max_ = 99.3%	*E*_max_ = 62.8%	*E*_max_ = 99.1%	

a Data expressed from three independent tests (*n* = 3), with a 95% confidence interval which is presented between “()”. *E*_max_ represents the maximum effect of the compound at its maximum concentration used.

Despite the difference in potency observed for compounds **9a**, **9b**, and **9d** in the biochemical assays against the PI3K isoforms and the mTOR enzyme (Table 2), they showed cytotoxic equipotency against the CRF-CEM and MOLT-4 cell lines. In these strains, there was no significant difference in cytotoxic activity between the dual PI3K/mTOR inhibitors (**9a** and **9d**) and the selective PI3K inhibitor (**9b**). Despite **9a** inhibits 11 times against PI3Kα isoform than **9b** and **9a**, the greatest cytotoxic efficacy, as measured by the maximum response (*E*_max_, Table 3), seems to depend on PI3Kδ inhibition, since **9d**, which inhibits PI3Kδ with an IC_50_ of 25.5 μM (approximately 42 times lower than the potency of **9a** and **9b** against this isoform) and mTOR with an IC_50_ of 2.5 μM, did not achieve a maximum response of more than 75%. A similar behavior for **9d** was observed in the MOLT-4 strain, which has mutations in PTEN and *PIK3R1*.

Analysis of the cytotoxic potency results in the MCF-7 solid tumor cell line, which has a mutation in the *PIK3CA* gene and therefore displays a PI3Kα activation phenotype, revealed that the difference in activity between compounds **9a** and **9b** did not exceed 2-fold. For compound **9a**, the maximum response observed was 63% at the highest concentration tested (100 μM). The lower potency of the compounds on MCF-7 solid line in relation to hematological neoplasms (Figure 4) can be attributed to the greater potency of **9b** and **9a** against PI3Kδ, the predominant isoform in hematopoietic cells.^33,34^

It can be seen, by comparing the data depicted in Tables 2 and 3, that the modifications introduced in hit **5f** did indeed lead to an increase in inhibitory potency over the target proteins but did not result in the optimization of cytotoxic potency in the phenotypic model used. **9a**, **9b**, and **9d** showed cytotoxic equipotency in relation to **5f**.

##### 3.3.2.2 Effect of **9a** against Drug-Resistant Lineage (K562 and Lucena Cells)

To evaluate the activity of compound **9a** against phenotypes of the resistant cell line, we chose another type of hematological cancer cell to compare whether its behavior would be like that of the parental and resistant cells. Thus, we conducted assays with compound **9a** on P53/*CDKN2A*-mutated K562 cells and its resistant variant, Lucena, using the MTT assay at 72 h. A concentration–response behavior was observed in the cells treated with **9a**. The analysis of the data exhibited in Figure 6 demonstrates the equipotency of compound **9a** in inhibiting both tumor lines, exhibiting an IC_50_ value of 9.44 μM on K562 and an IC_50_ value of 9.33 μM on Lucena (Figure 6 and Table 4). The ability of **9a** to reduce the cell viability of both parental and resistant cell lines suggests its potential for use in strains that express the multidrug-resistant (MDR) phenotype, which is the main barrier in Chronic Myelogenous Leukemia (CML) therapy.^35^ The ability to overcome the MDR phenotype is a multifactorial phenomenon, linked to different processes, such as epithelial-mesenchymal transition (EMT),^36–40^ and further studies need to be conducted to address this issue.

**Figure 6:**
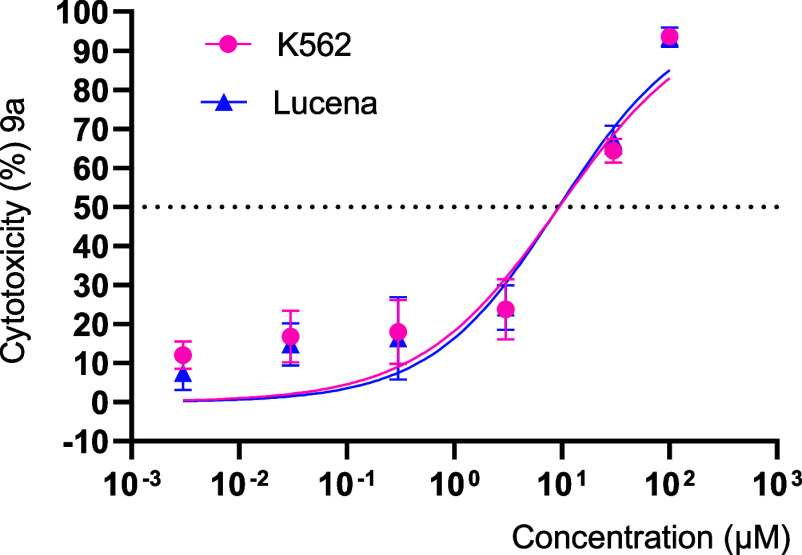
Cytotoxic evaluation of compound **9a** in K562 and Lucena cells treated with increasing concentrations (0.003–100 μM) for 72 h. After treatment, cell growth inhibition was monitored through the MTT assay, resulting in CC_50_ values of 9.438 and 9.331 μM, respectively. The results are presented as the percentage of toxicity of the treated cell population relative to the concentration of **9a**. Data presented as mean ± standard error of the mean of three independent experiments.

**Table 4:** Cytotoxic Concentration (CC_50_) of Compound **9a** against K562 and Lucena Cells, from 72 h Cell Viability Tests

Cell line	**9a** CC_50_ (μM)
K562	9.44 (5.02–17.75)
	*E*_max_ = 93.6%
Lucena	9.33 (5.48–15.90)
	*E*_max_ = 93.5%

Data were expressed from 3 independent tests (*n* = 3), with a 95% confidence interval which is presented between “()”. *E*_max_ represents the maximum effect of the compound at the maximum concentration used.

##### 3.3.2.3 Cytotoxic Effect of Compound **9a** on Human Peripheral Blood Mononuclear Cells (hPBMC)

To obtain information on the cytotoxic selectivity index of compound **9a**, its ability to interfere with hPBMC viability was studied using a 72 h MTT assay. As shown in Figure 7, at concentrations ranging from 0.02 to 50 μM, **9a** could not decrease hPBMC viability by more than 50%. At the highest concentration studied (50 μM), **9a** reduced viability by only 33%, showing a cytotoxic selectivity profile that favored tumor cell lines over nontumor cells. Gedatolisib was not cytotoxic to PBMCs, reducing the viability of normal mononuclear cells (*E*_max_ = 33%).^17^

**Figure 7:**
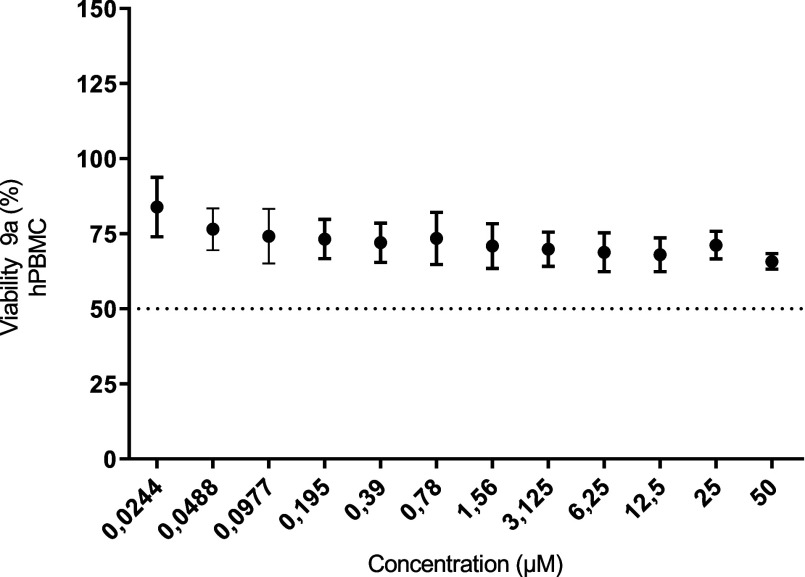
Evaluation of the cytotoxic action of compound **9a** in hPBMC by an MTT assay at 72 h. The assay was performed in the concentration range of 0.02–50 μM of **9a**. The data are representative of three independent experiments. *p* ≤ 0.05.

### 3.4 Permeability and Metabolic Stability

To understand the permeation profile of compound **9a**, a parallel artificial membrane permeability assay (PAMPA) was performed. As shown in Table 5, compound **9a** was insoluble in the PAMPA experiment using a membrane that mimics the blood–brain barrier, so it was not possible to determine its degree of permeation in this membrane.

**Table 5:** Permeability Coefficient of Compounds Using the PAMPA GIT and BBB Technique^a^

Compound	Pe. Exp. GIT (10^–6^ cm/s)	Fraction absorbed (%)	Classification GIT	Classification BBB	Pe. Exp. BBB (10^–6^ cm/s)	cLogP
9a	1.74	48.40	Medium	Insoluble		2.69

a cLogP values were calculated in silico by the ACD/Percepta program.

In PAMPA- and blood–brain barrier PAMPA (PAMPA-BBB) assays, experiments were conducted with **9a**, **9b**, and **9d**. As shown in Table 5, compound **9a** has a medium permeability in the PAMPA-GIT model. On the other hand, its azo-homologous analog with the isosteric exchange of the hydroxyl group for the amide group (**9d**) showed low permeability in the same model and this is due to the increase in the polarity of the molecule through the insertion of more polar groups confirmed by the decrease in cLogP shown in Table 6 which consequently resulted in a decrease in its permeability compared to compound **9a**. The permeability of compound **9b** could not be determined due to its insolubility.

**Table 6:** Microsomal Stability os **9a** in Rat Liver Microsomes

	With NADPH-generating system				Without NADPH-generating system				
Compounds	Metabolism Rate (%)	Elimination rate constant (*k*)	*t*_1/2_ (min)	Cl_int_ (mL/min/kg)	Metabolism Rate (%)	Elimination rate constant (*k*)	*t*_1/2_ (min)	Cl_int_ (mL/min/kg)	Recovery
9a	73.98	0.0202	34.3	0.86	5.34	0.0009	770	0.038	92.44

When the same compounds were performed in PAMPA-BBB, they all proved to be insoluble in this experiment, making it impossible to determine their permeability in this experimental model. It is worth noting that the solvent used in PAMPA-GIT, which uses the compound previously dissolved in DMSO, is different from PAMPA-BHE, which is carried out in ethanol, and that this change of solvent was a determining factor in the insolubility of the compounds in PAMPA-BHE. **9a** exhibited an absorption fraction in the PAMPA assay with a membrane that mimics the gastrointestinal tract (GIT) of 48.4%, being predicted as a compound of medium permeation through GIT (Table 5).

The metabolic stability of **9a** was also investigated, using rat liver microsomes (RLM) in the presence and absence of the NADPH-generating system. As shown in Table 6, **9a** had a half-life (*t*_1/2_) of 34.3 min in the presence of NADPH and a *t*_1/2_ of 770 min in its absence. Therefore, this suggests clearance through oxidative metabolism.

### 3.5 Docking Study

To predict the binding mode of compounds **9a**–**f** with PI3K and mTOR, docking analysis was performed using Gold v.2020.3. The X-ray crystal structure of PI3Kα (PDB ID: 4L23) and mTOR (PDB ID: 4JT6) was obtained as the starting point.^41,42^ The detailed binding modes of compounds **9a**, **9b**, and **9d** in the crystal structures of PI3Kα are illustrated in Figure 8. As expected, the oxygen atom of the morpholine ring formed a key hydrogen bond with Val851 in the hinge region of PI3Kα.

**Figure 8:**
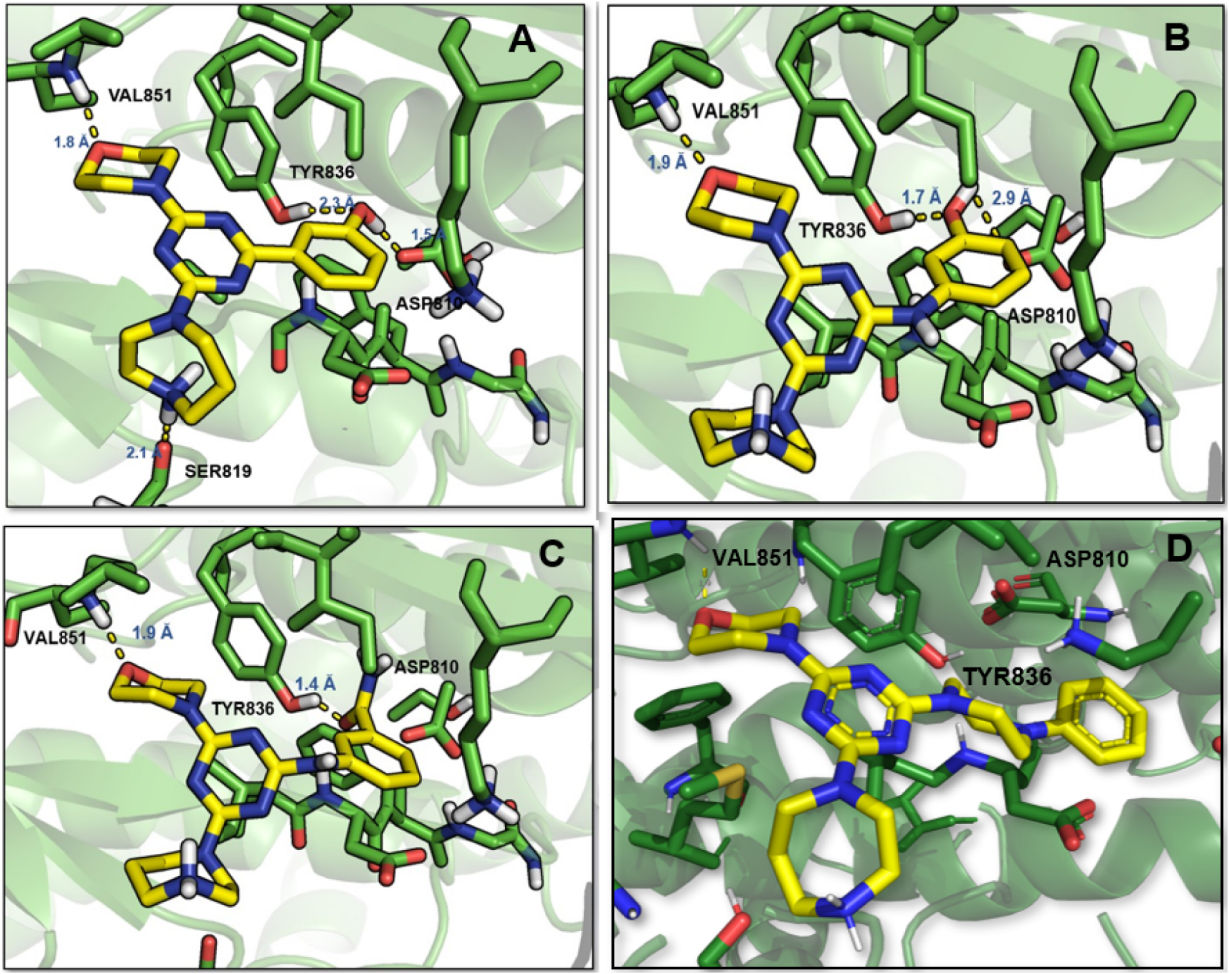
Binding mode for **9a** (A), **9b** (B), **9d** (C), and **5f** (D) into protein crystal structures of PI3Kα (PDB ID: 4L23).

Compounds **9a** and its aza-homologue **9b** exhibited two hydrogen bonds between the phenolic hydroxyl group and the residues Tyr836 and Asp810 in the affinity site (Figures 8A and 8B) of PI3Kα. Previous SAR studies describe the role of complementary interactions into the affinity site as essential to increase PI3K inhibitors’ potency.^41–43^ For compound **9a**, an additional interaction was observed between the hydrogen of the homopiperazine and the oxygen of Ser819 of PI3Kα (Figure 8A). The detailed mode of binding of **9d** revealed its ability to form an additional hydrogen bond between the carbonyl oxygen of the amide group and the Tyr836 residue in the affinity site of PI3Kα (Figure 8C).

Comparing these binding modes with the one performed by hit **5f**, it was possible to verify that only one hydrogen bond in the hinge region is realized by **5f**, involving the oxygen of the morpholine and the Val851 residue (Figure 8D). No hydrogen bonds were observed in the affinity site, which may explain the lower potency of **5f** in inhibiting PI3Kα.

The comparative binding mode of **9a**, **9d**, and **9e** with mTOR was studied and is illustrated in Figure 9. All compounds interact with the hinge region through a hydrogen bond between the oxygen of the morpholine and the Val2240 residue. The ability to make a hydrogen bond in the hinge domain is crucial for the inhibitory activity against both mTOR and PI3Kα.^43^ Like in PI3Kα, compound **9a** made hydrogen bonds at the affinity site of mTOR, involving phenolic hydroxyl with the Asp2195 and Tyr2225 residues (Figure 9A). **9d** was able to form a complementary hydrogen bond between the NH_2_ of the amide and the Asp2397 residue. For **9f**, no interaction in the affinity site of mTOR was observed; however, a hydrogen bond between the NH of the homopiperazine moiety and the Ser2342 residue was detected (Figure 9C), which may explain its lower inhibitory potency (Table 2).

**Figure 9:**
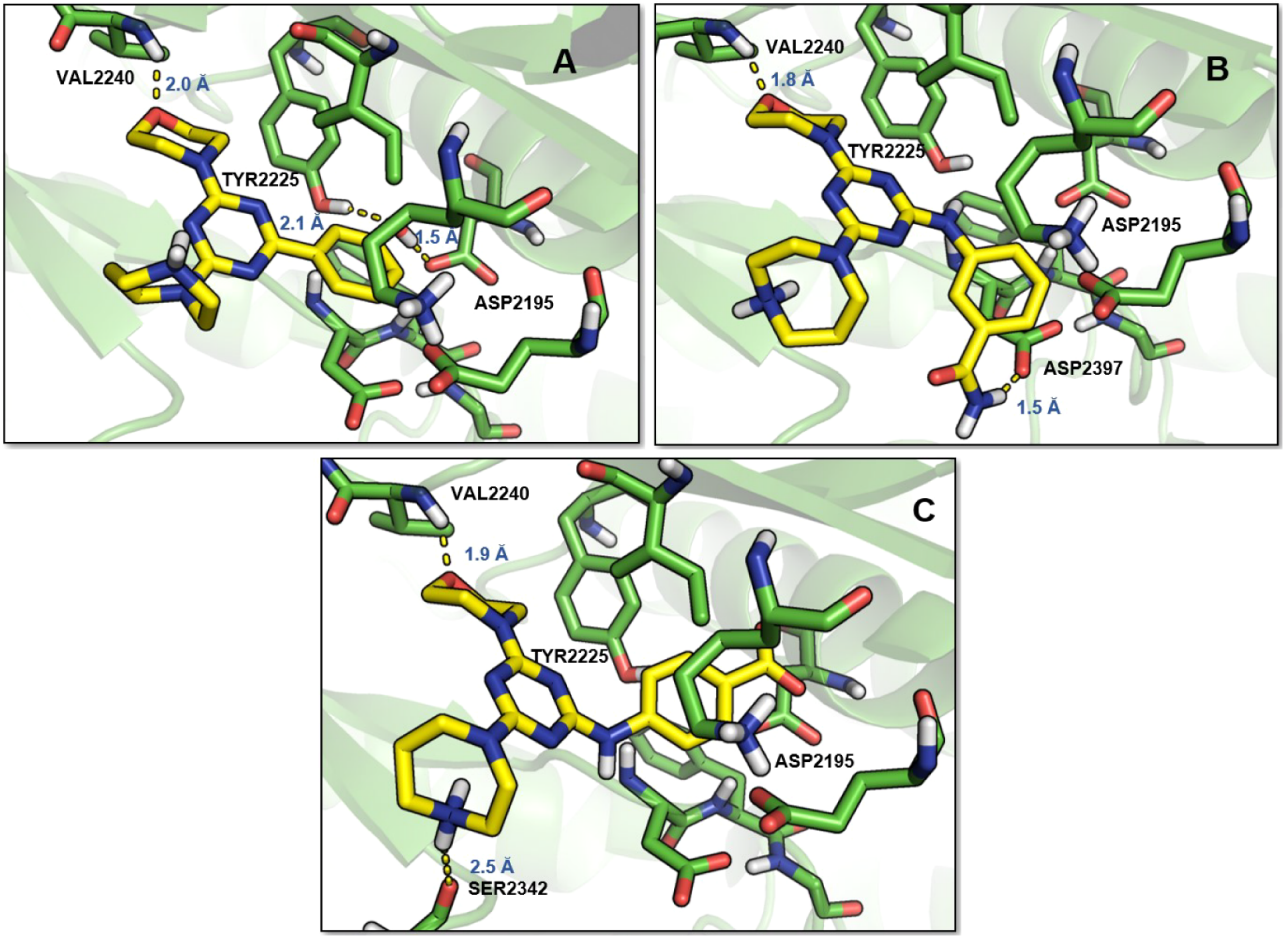
Binding mode for **9a** (A), **9d** (B), and **9e** (C) into protein crystal structures of mTOR (PDB ID: 4JT6).

## 4 Conclusions

In summary, our results allowed us to identify compound **9a** (LASSBio-2337) as a new dual Pan-PI3K/mTOR inhibitor, designed by structural modifications in hit **5f**. This inhibitor showed cytotoxic activity on different human leukemia cell lines, with potency ranging from 4.37 to 9.44 μM. However, it proved to be aqueous-insoluble, with average permeation in PAMPA-GIT and low metabolic stability in RLM, suggesting the need to optimize its DMPK properties.

## Supplementary Material



## Notes

PI3K and mTOR dose–response curves; calibration curves for kinetic solubility; rat liver microsomal stability assays with and without NADPH; detailed chemistry experimental procedures; synthesis and characterization of intermediates and final compounds; ^1^H and ^13^C NMR, HMQC, and HMBC spectra; HPLC purity traces; high-resolution mass spectrometry data (PDF)
