# Supplementary material for: New Dual Pan-PI3K/mTOR Inhibitor: Design, Synthesis, Cytotoxic Action, Permeation, Metabolic Stability, and In Silico Protein–Ligand Interaction
Source: ACS Omega. 2026 Feb 5;11(6):9719–33. doi: 10.1021/acsomega.5c10162 (PMC12917617; doi:10.1021/acsomega.5c10162)

Supporting Information

# New dual Pan-PI3K/mTOR inhibitor: design, synthesis, cytotoxic action, permeation, metabolic stability, and *in silico* protein-ligand interaction

Cristiane Aparecida e Silva [a, b], Raysa Magali Pillpe-Meza [a, b], Wesley Leandro Gouveia [a, b], Joana D'Arc da Silva Trindade [c], Gisele Barbosa [a], Amanda Marques Seixas Vieira [c], Heber Victor Tolomeu [a], Rayane França Pereira [c], Carlos Antônio do Nascimento Santos [c], Leonardo Freire-de-Lima [c], Lidia Moreira Lima [a, b, *]

[a] Laboratório de Avaliação e Síntese de Substâncias Bioativas (LASSBio), Instituto de Ciências Biomédicas, Universidade Federal do Rio de Janeiro, Avenida Carlos Chagas Filho, 373, Cidade Universitária, CEP 21941-902, Rio de Janeiro, RJ, Brasil.

[b] Programa de Pós-Graduação em Farmacologia e Química Medicinal, Instituto de Ciências Biomédicas, Universidade Federal do Rio de Janeiro, Avenida Carlos Chagas Filho, 373, Cidade Universitária, CEP 21941-902, Rio de Janeiro, RJ, Brasil.

[c] Laboratório de Glicobiologia (LABGLICO), Instituto de Biofísica Carlos Chagas Filho, Universidade Federal do Rio de Janeiro, Avenida Carlos Chagas Filho, 373, Cidade Universitária, CEP 21941-902, Rio de Janeiro, RJ, Brasil.

TABLE OF CONTENTS

**SUPPLEMENTARY FIGURES** .......... 4

**Figure S1. PI3K and mTOR dose-response curves** .......... 4

**Figure S2.** Calibration curves for determining the kinetic solubility of compounds 9a **(A)** 9b **(B)**, 9c **(C)**, 9d **(D)**, 9e **(E)**, 9f **(F)**. .......... 8

**Figure S3. A)** Rat liver microsomal stability profile of 9; **B)** First order rate constant (*k*) for elimination. Experiment carried out in the presence of a NADPH generating system. .......... 8

**Figure S4.** A) Rat liver microsomal stability profile of 9; B) First order rate constant (*k*) for elimination. Experiment carried out in the absence of a NADPH generating system. .......... 9

**CHEMISTRY** .......... 10

**Material and Methods** .......... 10

**Experimental Section** .......... 10

***Preparation of 4-(4,6-dichloro-1,3,5-triazin-2-yl) morpholine (7)*** .......... 10

***Preparation of 4-(4-chloro-6-(1,4-diazepan-1-yl)-1,3,5-triazin-2-yl) morpholine (8a)*** .......... 11

***Synthesis of 3-(4-(1,4-diazepan-1-yl)-6-morpholino-1,3,5-triazin-2-yl) phenol (9a)*** .......... 11

***General Procedure for Obtaining Disubstituted Triazines (8b-f)*** .......... 12

***General Procedure for obtaining the Final Compounds (9b-f)*** .......... 13

**$^{1}$H, $^{13}$C, HMQC and HMBC NMR SPECTRA OF FINAL COMPOUNDS** .......... 16

**Figure S5.** ***3-(4-(1,4-diazepan-1-yl)-6-morpholino-1,3,5-triazin-2-yl) phenol (9a)*** .16

**Figure S6.** ***3-((4-(1,4-diazepan-1-yl)-6-morpholino-1,3,5-triazin-2-yl)amino)phenol (9b)*** .......... 18

**Figure S7.** ***3-((4-(1,4-diazepan-1-yl)-6-morpholino-1,3,5-triazin-2-yl)amino)benzoic acid (9c)*** .......... 19

**Figure S8.** ***3-((4-(1,4-diazepan-1-yl)-6-morpholino-1,3,5-triazin-2-yl)amino)benzamide (9d)*** .......... 20

**Figure S9.** ***4-((4-(1,4-diazepan-1-yl)-6-morpholino-1,3,5-triazin-2-yl)amino)benzoic acid (9e)*** .......... 21

**Figure S10.** ***4-((4-(1,4-diazepan-1-yl)-6-morpholino-1,3,5-triazin-2-yl)amino)benzamide (9f)*** .......... 22

**HPLC TRACES OF FINAL COMPOUNDS** .......... 23

**Figure S11.** ***3-(4-(1,4-diazepan-1-yl)-6-morpholino-1,3,5-triazin-2-yl) phenol (9a)*** 23

**Figure S12.** ***3-((4-(1,4-diazepan-1-yl)-6-morpholino-1,3,5-triazin-2-yl)amino)phenol (9b)*** .......... 24

**Figure S13.** ***3-((4-(1,4-diazepan-1-yl)-6-morpholino-1,3,5-triazin-2-yl)amino)benzoic acid (9c)*** .......... 25

**Figure S14.** ***3-((4-(1,4-diazepan-1-yl)-6-morpholino-1,3,5-triazin-2-yl)amino)benzamide (9d)*** ....26
**Figure S15.** ***4-((4-(1,4-diazepan-1-yl)-6-morpholino-1,3,5-triazin-2-yl)amino)benzoic acid (9e)*** ....27
**Figure S16.** ***4-((4-(1,4-diazepan-1-yl)-6-morpholino-1,3,5-triazin-2-yl)amino)benzamide (9f)*** ....28
**MASS SPECTROMETRY DATA** ....29
**Figure S17.** ***Synthesis of 3-(4-(1,4-diazepan-1-yl)-6-morpholino-1,3,5-triazin-2-yl) phenol (9a)*** ....29
**Figure S18.** ***3-((4-(1,4-diazepan-1-yl)-6-morpholino-1,3,5-triazin-2-yl)amino)phenol (9b)*** ....29
**Figure S19.** ***3-((4-(1,4-diazepan-1-yl)-6-morpholino-1,3,5-triazin-2-yl)amino)benzoic acid (9c)*** ....30
**Figure S20.** ***3-((4-(1,4-diazepan-1-yl)-6-morpholino-1,3,5-triazin-2-yl)amino)benzamide (9d)*** ....30
**Figure S21.** ***4-((4-(1,4-diazepan-1-yl)-6-morpholino-1,3,5-triazin-2-yl)amino)benzoic acid (9e)*** ....31
**Figure S22.** ***4-((4-(1,4-diazepan-1-yl)-6-morpholino-1,3,5-triazin-2-yl)amino)benzamide (9f)*** ....32

**SUPPLEMENTARY FIGURES**

**Figure S1. PI3K and mTOR dose-response curves**

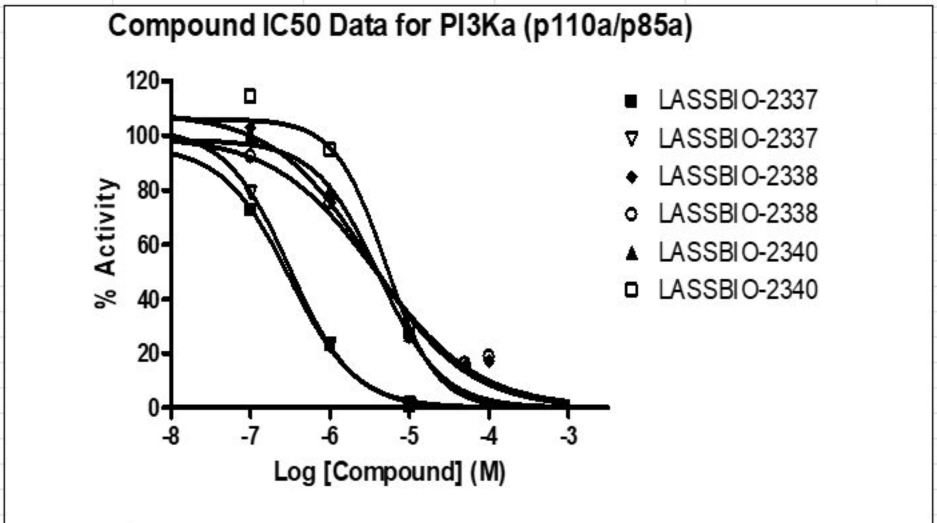


| | LASSBIO-2337 | LASSBIO-2337 | LASSBIO-2338 | LASSBIO-2338 | LASSBIO-2340 | LASSBIO-2340 |
|---|---|---|---|---|---|---|
| HILLSLOPE | -1.034 | -1.082 | -0.7108 | -0.6732 | -1.093 | -1.369 |
| EC50 | 3.060e-007 | 3.112e-007 | 3.173e-006 | 3.815e-006 | 4.056e-006 | 4.996e-006 |

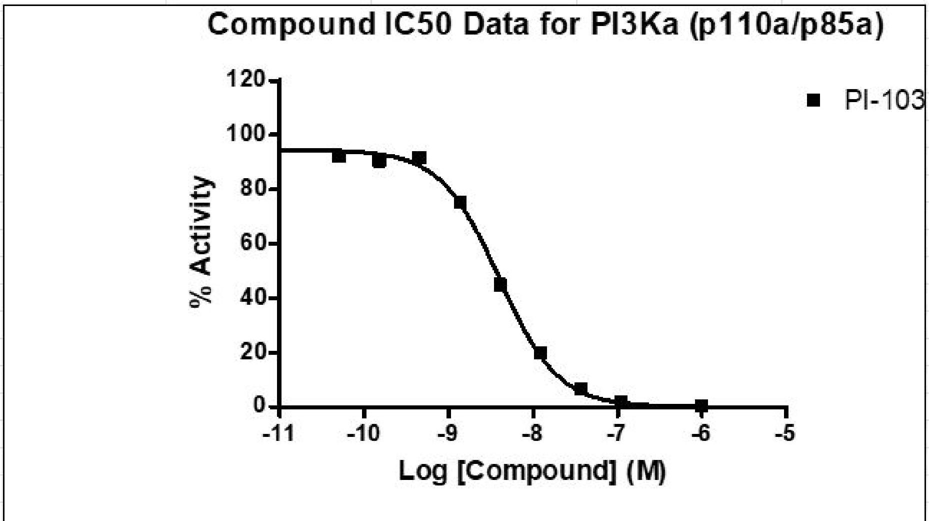


| | PI-103 |
|---|---|
| HILLSLOPE | -1.244 |
| EC50 | 3.972e-009 |

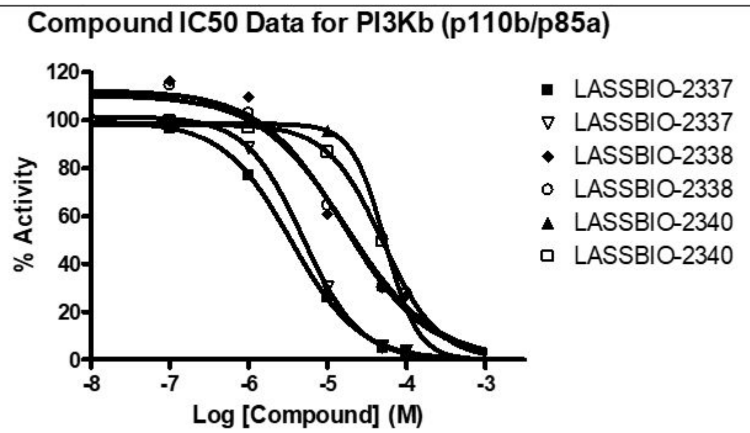


| | LASSBIO-2337 | LASSBIO-2337 | LASSBIO-2338 | LASSBIO-2338 | LASSBIO-2340 | LASSBIO-2340 |
|---|---|---|---|---|---|---|
| HILLSLOPE | -1.006 | -1.204 | -0.8185 | -0.7896 | -2.070 | -1.238 |
| EC50 | 3.445e-006 | 4.900e-006 | 1.599e-005 | 1.734e-005 | 5.369e-005 | 5.058e-005 |

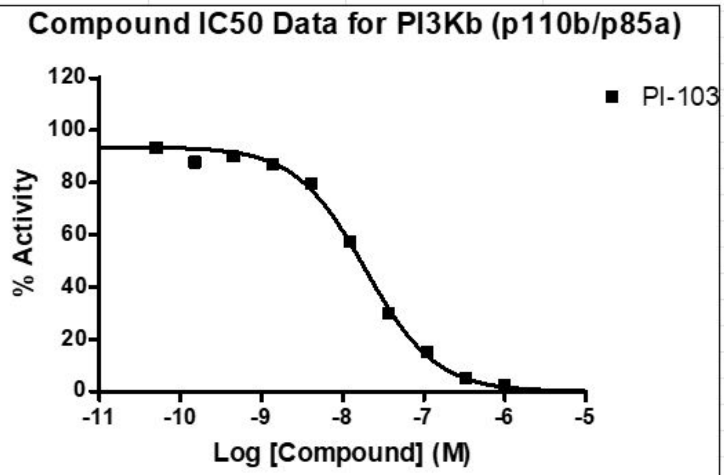


| | PI-103 |
|---|---|
| HILLSLOPE | -1.006 |
| EC50 | 1.899e-008 |

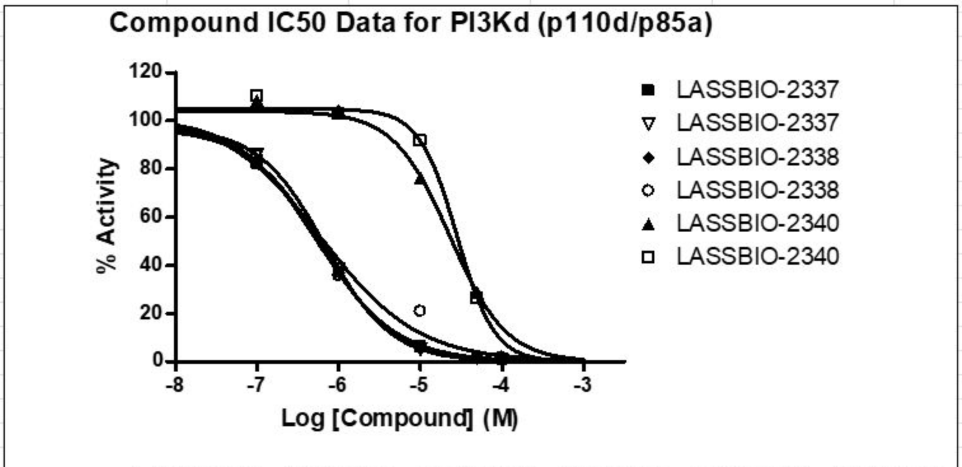


| | LASSBIO-2337 | LASSBIO-2337 | LASSBIO-2338 | LASSBIO-2338 | LASSBIO-2340 | LASSBIO-2340 |
|---|---|---|---|---|---|---|
| HILLSLOPE | -0.8955 | -1.059 | -0.9651 | -0.7272 | -1.259 | -1.886 |
| EC50 | 5.352e-007 | 6.724e-007 | 6.093e-007 | 6.067e-007 | 2.303e-005 | 2.800e-005 |

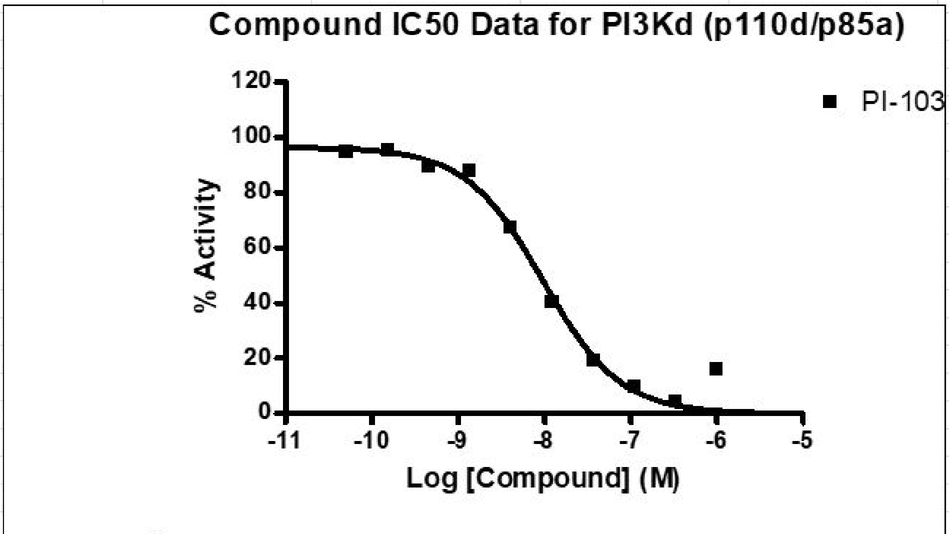


| | PI-103 |
|---|---|
| HILLSLOPE | -0.9704 |
| EC50 | 9.609e-009 |

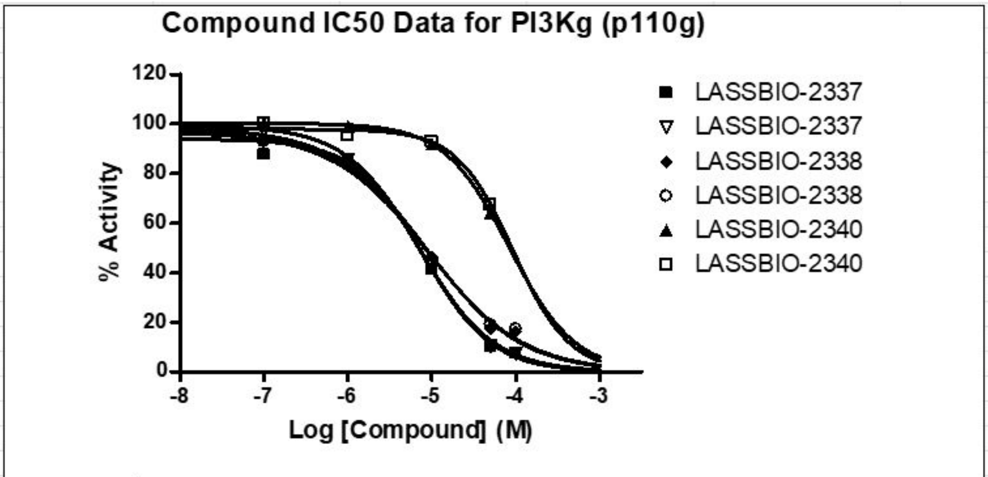


| | LASSBIO-2337 | LASSBIO-2337 | LASSBIO-2338 | LASSBIO-2338 | LASSBIO-2340 | LASSBIO-2340 |
|---|---|---|---|---|---|---|
| HILLSLOPE | -1.056 | -0.9836 | -0.7812 | -0.7639 | -1.116 | -1.292 |
| EC50 | 7.906e-006 | 7.168e-006 | 8.812e-006 | 8.951e-006 | 8.419e-005 | 9.283e-005 |

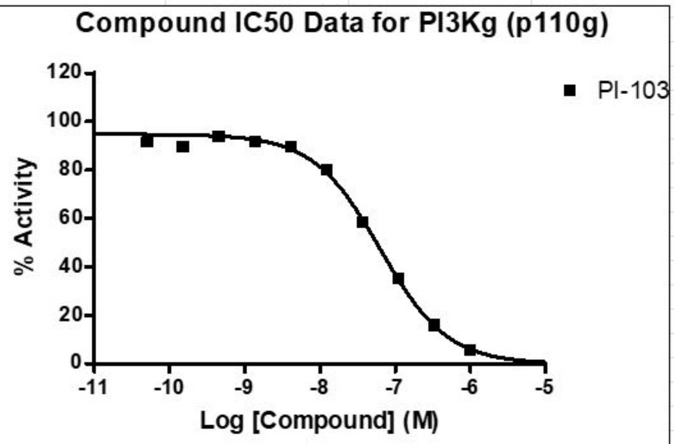


| | PI-103 |
|---|---|
| HILLSLOPE | -0.9764 |
| EC50 | 6.303e-008 |

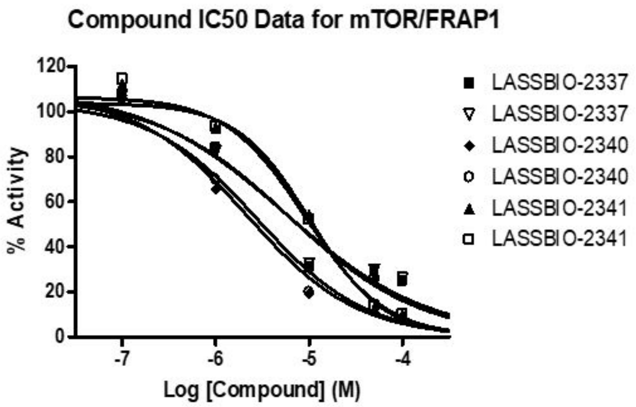


| | LASSBIO-2337 | LASSBIO-2337 | LASSBIO-2340 | LASSBIO-2340 | LASSBIO-2341 | LASSBIO-2341 |
|---|---|---|---|---|---|---|
| HILLSLOPE | -0.6174 | -0.5695 | -0.7395 | -0.7502 | -1.106 | -1.016 |
| EC50 | 6.122e-006 | 5.519e-006 | 2.168e-006 | 2.816e-006 | 1.028e-005 | 9.137e-006 |

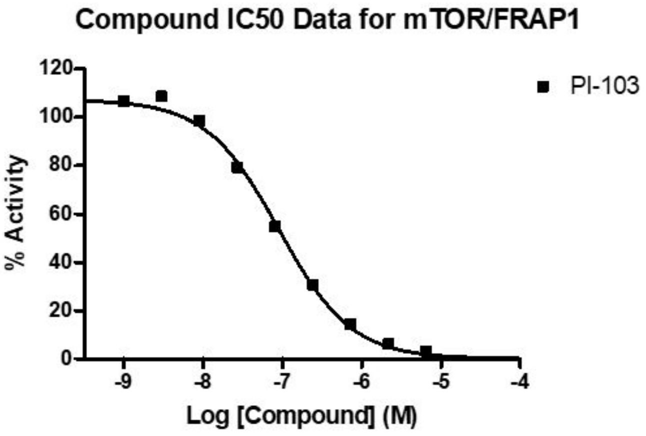


| | PI-103 |
|---|---|
| HILLSLOPE | -0.9501 |
| EC50 | 9.028e-008 |

**Figure S2.** Calibration curves for determining the kinetic solubility of compounds 9a **(A)** 9b **(B)**, 9c **(C)**, 9d **(D)**, 9e **(E)**, 9f **(F)**.

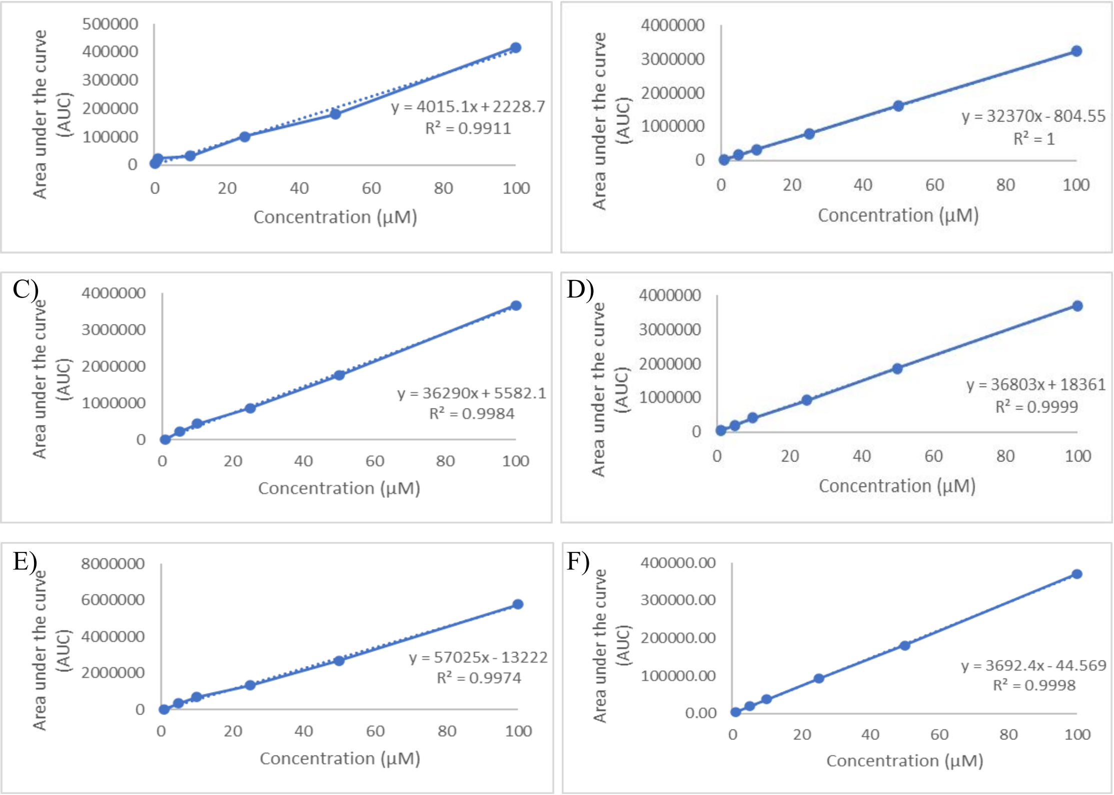


**Figure S3.** **A)** Rat liver microsomal stability profile of 9; **B)** First order rate constant (*k*) for elimination. Experiment carried out in the presence of a NADPH generating system.

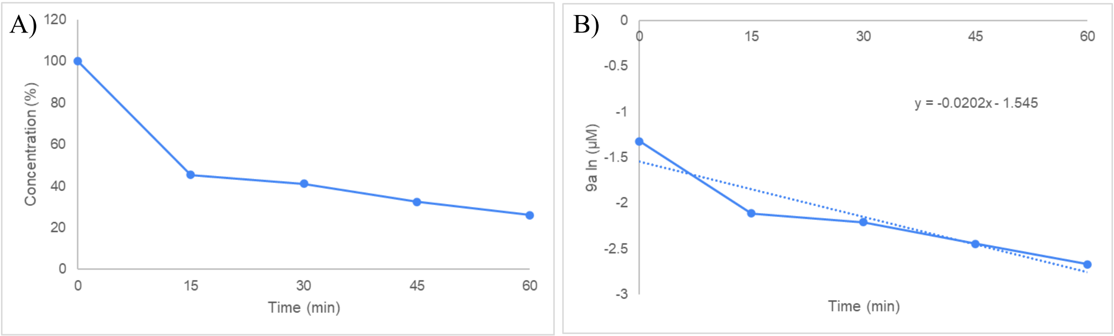

**Figure S4.** A) Rat liver microsomal stability profile of 9; B) First order rate constant (*k*) for elimination. Experiment carried out in the absence of a NADPH generating system.

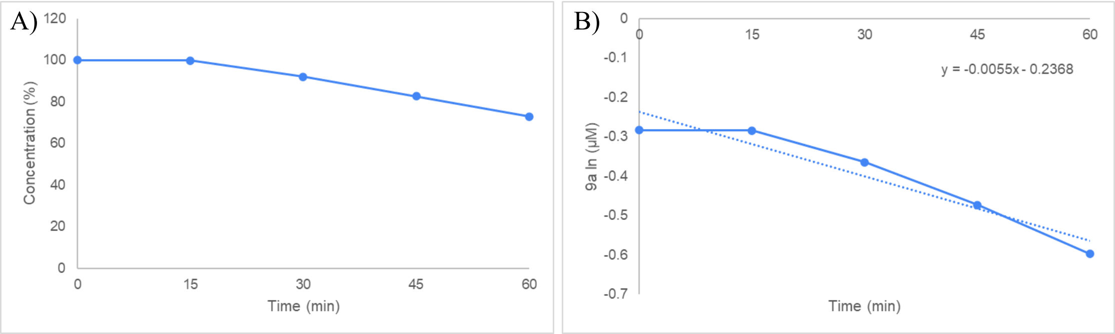

## CHEMISTRY

### Material and Methods

All solvents and reagents obtained from commercial sources were used without further purification. Flash column chromatography was performed using silica (200-300 mesh). Reactions run at elevated temperatures were carried out in the oil bath. All reactions were monitored by thin-layer chromatography. $^1$H NMR and $^{13}$C NMR spectra were recorded on a Bruker AV 500 or Variam 400-MR spectrometer and were calibrated using TMS or residual deuterated solvent as an internal reference ($CDCl_3$:$^1$H, δ = 7.26 ppm; DMSO-$_{d6}$: δ= 2.50 ppm, acetic acid-$_{d4}$: δ = 2.03, 11.53 ppm). The purity of the synthesized compounds was evaluated using a high-performance liquid chromatography (SHIMADZU, LC-20AD 3D) equipped with a Kromasil 100-5C18, and the purity of the biologically tested compounds was ≥ 95%.

$^1$H NMR spectral data are reported in terms of chemical shift (δ, ppm), multiplicity, coupling constant (Hz), and integration. $^{13}$C NMR spectral data are reported in terms of chemical shift (d, ppm) and multiplicity. Peaks were labeled as singlet (s), doublet (d), triplet (t), quartet (q), and multiplet (m). High resolution mass spectra were performed on QExactive Hybrid Quadrupole Orbitrap mass spectrometer.

### Experimental Section

***Preparation of 4-(4,6-dichloro-1,3,5-triazin-2-yl) morpholine (7)***

A solution containing triethylamine (1.0 mL, 7.33 mmol) and morpholine (0.6 mL, 7.4 mmol) in acetonitrile was added dropwise to an acetonitrile solution containing cyanuric chloride (6) (1.5 g, 8.15 mmol). The mixture was stirred in an ice bath for two hours. After the reaction was complete, the organic solvent was concentrated using a rotary

evaporator, and distilled water was added to the crude product, followed by vacuum filtration. The obtained material was purified by recrystallization from an acetone/water mixture, yielding the final product (**7**) with an 83% yield.

$^{1}$H NMR (400 MHz, CDCl3) δ 3.81 (t, *J*=4, 4H); 3.67 (t, *J*=4 Hz,4H). $^{13}$C NMR (100 MHz, $CDCl_3$*)* δ 170.45, 164.17, 66.32, 44.47.

***Preparation of 4-(4-chloro-6-(1,4-diazepan-1-yl)-1,3,5-triazin-2-yl) morpholine (8a)***

To a solution containing 4-(4,6-dichloro-1,3,5-triazin-2-yl) morpholine (**7**) in acetonitrile (15 mL) was added a saturated NaHCO3 solution (1:1) and 1 equivalent of homopiperazine. The reaction was stirred at room temperature, and upon completion, the organic solvent was concentrated. The solid formed in the aqueous phase was filtered under vacuum, yielding the final product (**8a**) with a 70% yield. $^{1}$H NMR (400 MHz, CDCl3) δ 3.81 (t, *J*=4, 4H); 3.67 (t, *J*=4 Hz,4H). $^{13}$C NMR (100 MHz, $CDCl_3$*)* δ 170.45, 164.17, 66.32, 44.47.

***Synthesis of 3-(4-(1,4-diazepan-1-yl)-6-morpholino-1,3,5-triazin-2-yl) phenol (9a)***

To a solution of 4-(4-chloro-6-(1,4-diazepan-1-yl)-1,3,5-triazin-2-yl) morpholine (**8**) (0.5 g; 1.7 mmol) in 50 mL of an acetonitrile/water mixture (1:1) was added $Na_2CO_3$ (0.7 g, 6.8 mmol), 3-hydroxyphenylboronic acid (0.2 g, 1.7 mmol), and the palladium catalyst $PdCl_2(PPh3)_2$ (0.05 g). The reaction mixture was stirred magnetically at 200°C, and after four hours, the reaction was observed to be complete. The organic solvent was concentrated, resulting in a precipitate in the aqueous phase which was filtered under vacuum and purified by column chromatography to obtain the final product with a 70% yield.$^{1}$H NMR (500 MHz- DMSO – $d_6$*)* δ 9.95-9.52 (m, 1H), 7.78-7.73 (m,2H), 7.28-7.16 (m, 1H), 6.94-6.89 (m, 1H).$^{13}$C (125 MHz- DMSO – $d_6$*)* δ 169.58, 164.91,164.70, 157.43,

138.64, 129.50, 119.15, 118.64, 114.94, 66.17, 46.69, 46.05, 45.62, 45.44, 43.41, 25.67.HRMS (ESI): m/z [M+H]$^{+}$ calcd. for $[C_{18}H_{24}N_6O_2]^{+}$:357.1994, found: 357.2029

***General Procedure for Obtaining Disubstituted Triazines (8b-f)***

To a solution containing 1 equivalent of 4-(4,6-dichloro-1,3,5-triazin-2-yl) morpholine (**7**) in acetonitrile was added a saturated NaHCO3 solution (1:1) and 1.5 equivalents of the appropriate aniline. The reaction was stirred at room temperature, and upon completion, the organic solvent was concentrated. The solid formed in the aqueous phase was filtered and purified by recrystallization from hot ethanol, characterized, and used in subsequent steps.

*3-((4-chloro-6-morpholino-1,3,5-triazin-2-yl)amino)phenol* ***(8b)*** 83% isolated yield, white solid; $^{1}$H NMR (500 MHz- DMSO – *d*$_6$) δ 9.95 (sl, H-1H), 9.48 (s, 1H), 7.16 (s, 1H), 7.08 (t, *J*=8, 1H), 7.03-7.01 (m, 1H) 6.45 (dd, *J*= 8 Hz, *J*=2 Hz, 1H), 3.75-3.50 (m, H-4, 8H);$^{13}$C NMR (125 MHz- DMSO – *d*$_6$) δ 168.48, 164.30, 163.51, 157.60, 139.79, 129.49, 111.22, 110.52, 107.52, 65.93, 43.86.

*3-((4-chloro-6-morpholino-1,3,5-triazin-2-yl)amino)benzoic acid* ***(8c)*** 75% isolated yield, white solid. $^{1}$H NMR (500 MHz- DMSO – *d*$_6$) δ 9.97 (s, 1H), 8.44 (s, 1H), 7.76 (dd, *J* = 8, *J* = 2, 1H), 7.62 (d, *J* = 8, 1H), 7.42 (t, *J* = 8 Hz, 1H), 3.82 – 3.72 (m, 4H), 3.69 – 3.64 (m,4H). $^{13}$C NMR (125 MHz- DMSO – *d*$_6$) δ 172.15, 167.24, 164.11,139.07, 131.24, 128.94, 124.18, 123.82, 121.10, 65.72, 43.84.

*3-((4-chloro-6-morpholino-1,3,5-triazin-2-yl)amino)benzamide* ***(8d)*** 85% isolated yield, white solid; $^{1}$H NMR (500 MHz- DMSO – *d*$_6$) δ 10.24 (s, 1H), 8.33 (s, 1H), 7.92 (s, 1H), 7.70 (d, *J*=8 Hz,1 H), 7.54 (d, *J*=8 Hz, 1H), 7.38 (t, *J*=8 Hz, 1H), 7.33 (s, 1H), 3.79 (s, 1H), 3.72 (s, 2H), 3.69-3.62 (m, 4H); $^{13}$C NMR (125 MHz- DMSO – *d*$_6$) δ 168.55, 167.89,164.09, 163.52, 138.76, 134.96, 128.45, 122.94, 121.84, 120.11, 65.72, 43.77.

*4-((4-chloro-6-morpholino-1,3,5-triazin-2-yl)amino)benzoic acid **(8e)*** 80% isolated yield, white solid; $^1$H NMR (400 MHz- DMSO – $d_6$) δ 10.07 (s, 1H), 7.89 (d, 2H), 7.75 (d, 2H), 3.77 – 3.74 (m, 4H), 3.68 – 3.66 (m,4H); $^{13}$C NMR (100 MHz- DMSO – $d_6$) δ 168.75, 167.17, 164.22, 163.67, 143.06, 130.45, 124.92, 119.60, 65.90, 43.93.

*4-((4-chloro-6-morpholino-1,3,5-triazin-2-yl)amino)benzamide **(8f)*** 88% isolated yield, white solid; $^1$H NMR (400 MHz- DMSO – $d_6$) δ 10.29 (s, 1H),7.87 (s, 1H), 7.84 (d, $J$ = 8 Hz,2H), 7.70 (d, $J$ = 8 Hz, 2H), 7.23 (s, 1H), 3.78 – 3.74 (m, 2H), 3.73 – 3.69 (m, 2H), 3.69 – 3.65 (m, 2H), 3.64 (d, $J$ = 4 Hz,2H); $^{13}$C NMR (100 MHz- DMSO – $d_6$) 168.69,167.69, 164.23, 163.62, 141.58, 128.55, 128.44, 119.43, 65.90, 65.80, 44.00, 43.87.

***General Procedure for obtaining the Final Compounds (9b-f)***

To a mixture containing 1 equivalent of the disubstituted intermediate (**8b-f**) in dioxane/water (1:1), 1.5 equivalents of homopiperazine and 2.5 equivalents of Na2CO3 were added. The resulting mixture was stirred and refluxed, and the reaction was observed to be complete after 6 hours. The solution was concentrated under vacuum, and the crude product was purified by column chromatography on silica gel (dichloromethane/methanol = 10:1).

*3-((4-(1,4-diazepan-1-yl)-6-morpholino-1,3,5-triazin-2-yl)amino)phenol **(9b)*** 60% isolated yield, white solid; HPLC purity 99.2%; M.p: 215-217 ºC; $^1$H NMR (500 MHz- DMSO – $d_6$) δ 11.02 (s, 1H), 10.67 (s, 1H), 7.18 (t, $J$ = 8 Hz, 1H), 7.00 (t, $J$ =2.2, 1H), 6.91 (ddd, $J$ = 8, $J$ =2 Hz,1H), 6.63 (ddd, $J$ =8, $J$ = 2 Hz, 1H), 3.80 – 3.61 (m, 16H), 3.11- 3.06 (m, 2H); $^{13}$C NMR (125 MHz- DMSO – $d_6$) δ 164.72, 164.44, 164.00,157.35, 141.57, 128.87, 110.36, 108.58, 106.61, 66.01, 65.87, 62.05, 47.21, 46.47, 43.32, 25.50; HRMS (ESI): m/z $[M+H]^+$ calcd. for $[C_{18}H_{25}N_7O_2]^+$:372.2103, found: 372.2147.

*3-((4-(1,4-diazepan-1-yl)-6-morpholino-1,3,5-triazin-2-yl)amino)benzoic acid* ***(9c)*** 50% isolated yield, white solid; HPLC purity 99.7%; M.p: 250-252 ºC; $^1$H NMR (500 MHz- DMSO – *d₆)* δ 9.43 (1H,s, H-22), 8.95 (s,1H), 8.58 (s,1H), 7.67 (d, *J* = 8 Hz,1H), 7.55 (d, *J* = 8 Hz,1H), 7.38 (t, J = 8 Hz,1H), 4.02 – 3.95 (m, 2H), 3.87 – 3.80 (m, 2H), 3.78 – 3.68 (m, 4H), 3.68 – 3.60 (m, 4H), 3.27 (s, 2H), 3.18 (s,2H), 2.09-204 (m, 2H); $^{13}$C NMR (125 MHz- DMSO – $d_6$) δ 167.40, 167.29, 163.24, 162.10, 139.78, 131.21, 128.72, 123.76, 123.03, 120.75, 65.94, 45.14,44.82, 44.72, 43.80, 42.75, 24.84; HRMS (ESI): m/z $[M+H]^+$ calcd. for $[C_{19}H_{25}N_7O_3]^+$:400.2052, found: 400.2083.

*3-((4-(1,4-diazepan-1-yl)-6-morpholino-1,3,5-triazin-2-yl)amino)benzamide* ***(9d)*** 65% isolated yield, white solid; HPLC purity 98.6%; M.p: 280-282 ºC; $^1$H NMR (400 MHz- DMSO – $d_6$) δ 8.85 (s, 1H), 8.38 (s, 1H), 7.71 (d, *J* = 8 Hz, 1H), 7.41 (d, *J* = 8 Hz, 1H), 7.28 (t, *J* = 8 Hz, 3H), 4.10 – 3.40 (m, 16H), 1.94 (q, *J* = 11, *J* = 8 Hz, 2H); $^{13}$C NMR (100 MHz- DMSO – $d_6$) δ 168.46, 164.73, 164.52, 164.07, 140.58, 134.87, 128.07, 121.97, 120.15, 119.07, 66.03, 47.33, 46.15, 45.77, 45.22, 43.32, 30.74; HRMS (ESI): m/z $[M+H]^+$ calcd. for $[C_{19}H_{26}N_8O_2]^+$:399.2212, found: 399.2241.

*4-((4-(1,4-diazepan-1-yl)-6-morpholino-1,3,5-triazin-2-yl)amino)benzoic acid* ***(9e)*** 60% isolated yield, white solid; HPLC purity 98.4%; M.p: 218-220 ºC **;** $^1$H NMR (500 MHz- Ácido acético – $d_4$) δ 8.04 (d, *J* = 8 Hz, 2H), 7.79 (d, *J* = 8 Hz, 2H), 4.13 (t, *J* = 5 Hz, 2H), 3.95 (t, *J* = 6 Hz, 2H), 3.87 – 3.78 (m, H-6, 8H), 3.55 (t, *J* = 5 Hz, 2H), 3.43 (t, *J*=5 Hz,2H), 2.28-2.23 (m, 2H); $^{13}$C NMR (125 MHz- Ácido acético – $d_4$) δ 170.54, 163.96, 163.57,163.57,144.29, 130.96,123.25 (C-20),119.25, 66.19,46.05, 45.62, 45.00, 44.02,42.90, 24.92; HRMS (ESI): m/z $[M+H]^+$ calcd. for $[C_{19}H_{25}N_7O_3]^+$:400.2052, found: 400.2089.

*4-((4-(1,4-diazepan-1-yl)-6-morpholino-1,3,5-triazin-2-yl)amino)benzamide* ***(9f)*** 70% isolated yield, white solid; HPLC purity 98.5%; M.p: 300 ºC; $^1$H NMR (400 MHz-

Ácido acético – *$d_4$)* δ 7.89 (d, *J* = 8 ,2H), 7.76 (d, *J* = 8 Hz,2H), 4.13 ( t, *J*=5 Hz, 2H), 3.96 (t, *J* = 6 Hz, 2H), 3.88 – 3.82 (m, 4H), 3.80 (d, *J* = 5 Hz, 4H), 3.55(t, *J*=5 Hz, 2H), 3.43 (t, *J*= 5 Hz, 2H), 2.28-2.22 (m,2H), 1.33 (sl,1H); $^{13}$C NMR (100 MHz- DMSO – *$d_6$)* δ 167.60, 164.68, 164.45, 163.92, 143.33, 128.12, 126.69, 118.16, 65.99, 46.49, 46,22 , 45.76 , 45.08, 43.36, 25.72;HRMS (ESI): m/z $[M+H]^+$ calcd. for $[C_{19}H_{26}N_8O_2]^+$:399.2212, found: 399.2249.

**$^1$H, $^{13}$C, HMQC and HMBC NMR SPECTRA OF FINAL COMPOUNDS**

**Figure S5.** *3-(4-(1,4-diazepan-1-yl)-6-morpholino-1,3,5-triazin-2-yl) phenol (9a)*

$^1$H-NMR

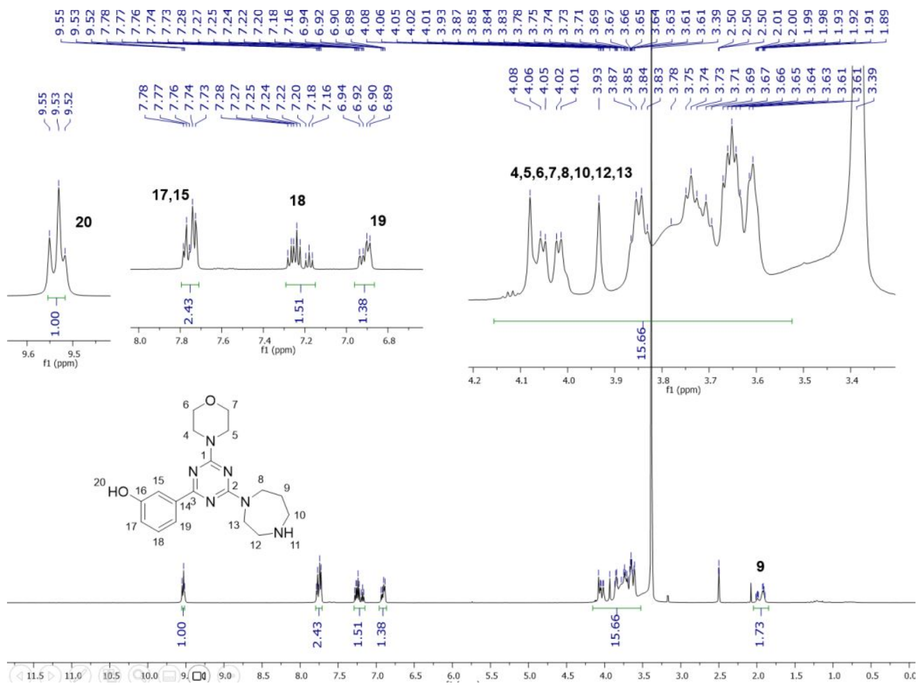

$^{13}$C-NMR

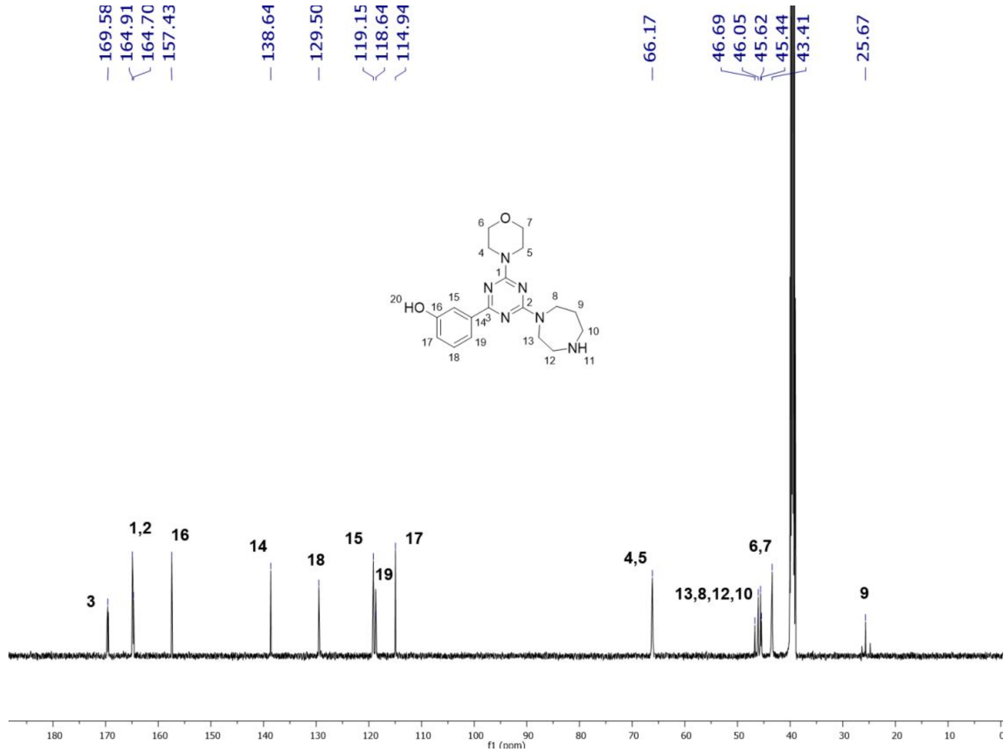

HMQC

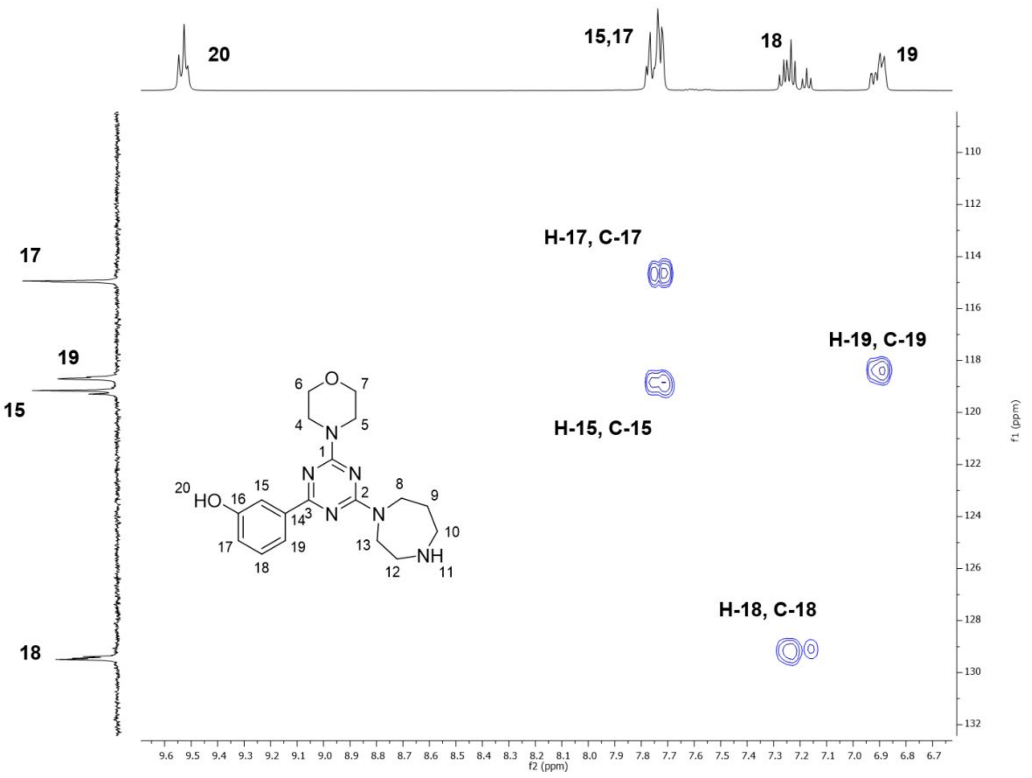

20
15,17
18
19
17
19
15
18
H-17, C-17
H-19, C-19
H-15, C-15
H-18, C-18
f2 (ppm)
f1 (ppm)


HMBC

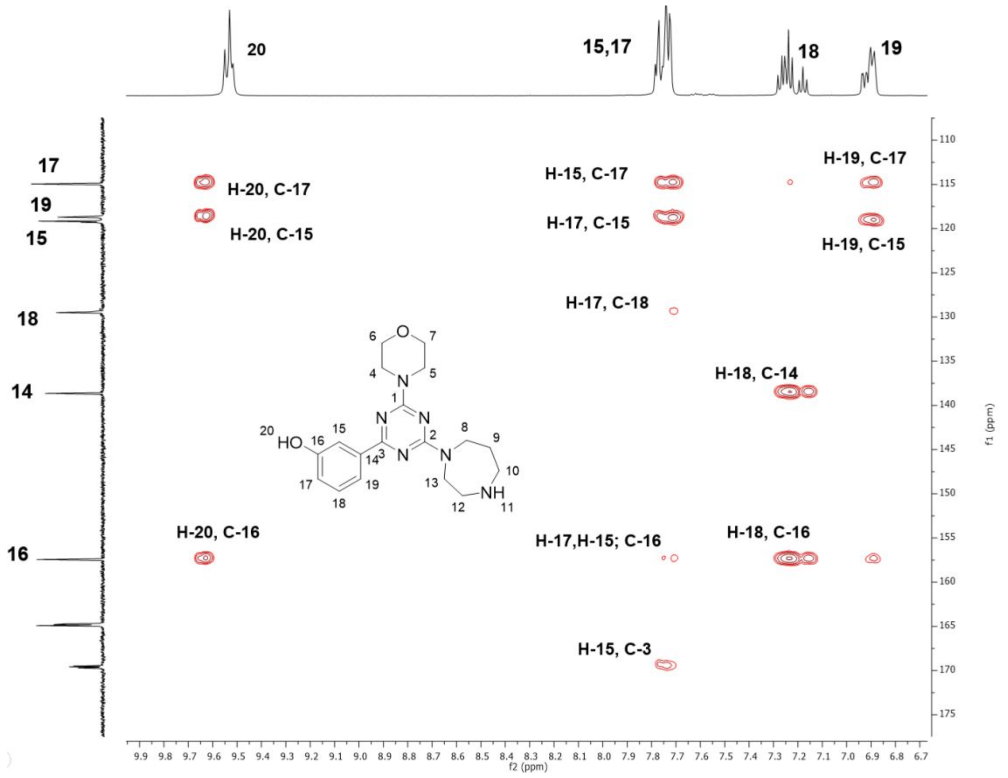

20
15,17
18
19
17
19
15
18
14
16
H-20, C-17
H-20, C-15
H-15, C-17
H-17, C-15
H-19, C-17
H-19, C-15
H-17, C-18
H-18, C-14
H-20, C-16
H-17,H-15; C-16
H-18, C-16
H-15, C-3
f2 (ppm)
f1 (ppm)

**Figure S6.** ***3-((4-(1,4-diazepan-1-yl)-6-morpholino-1,3,5-triazin-2-yl)amino)phenol (9b)***

$^{1}$H-NMR

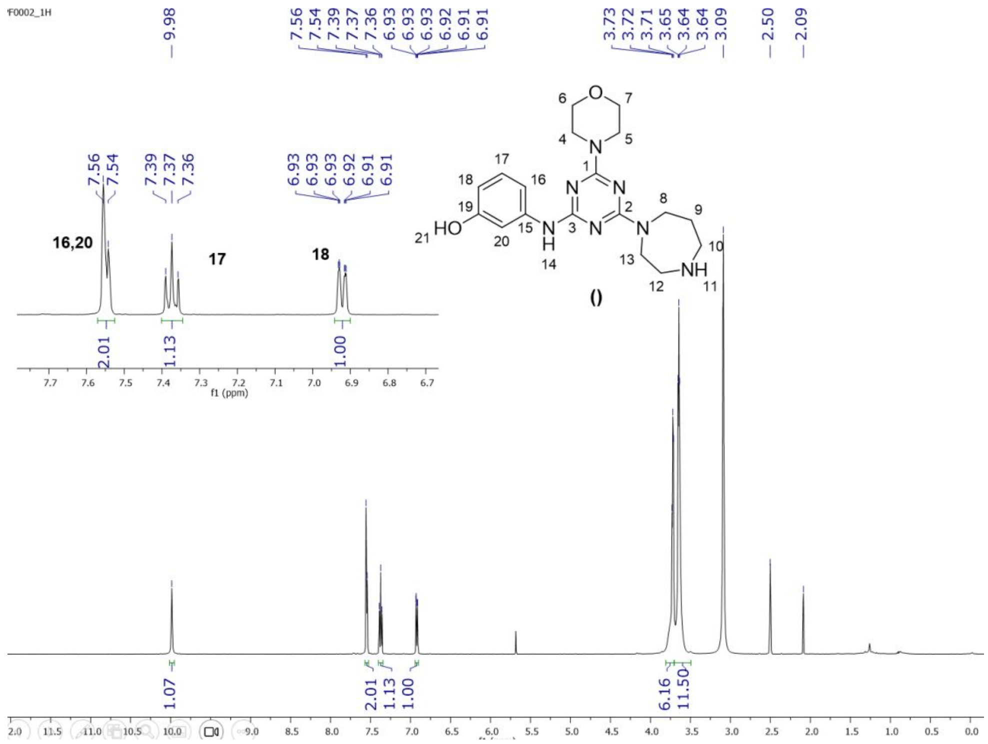


$^{13}$C-NMR

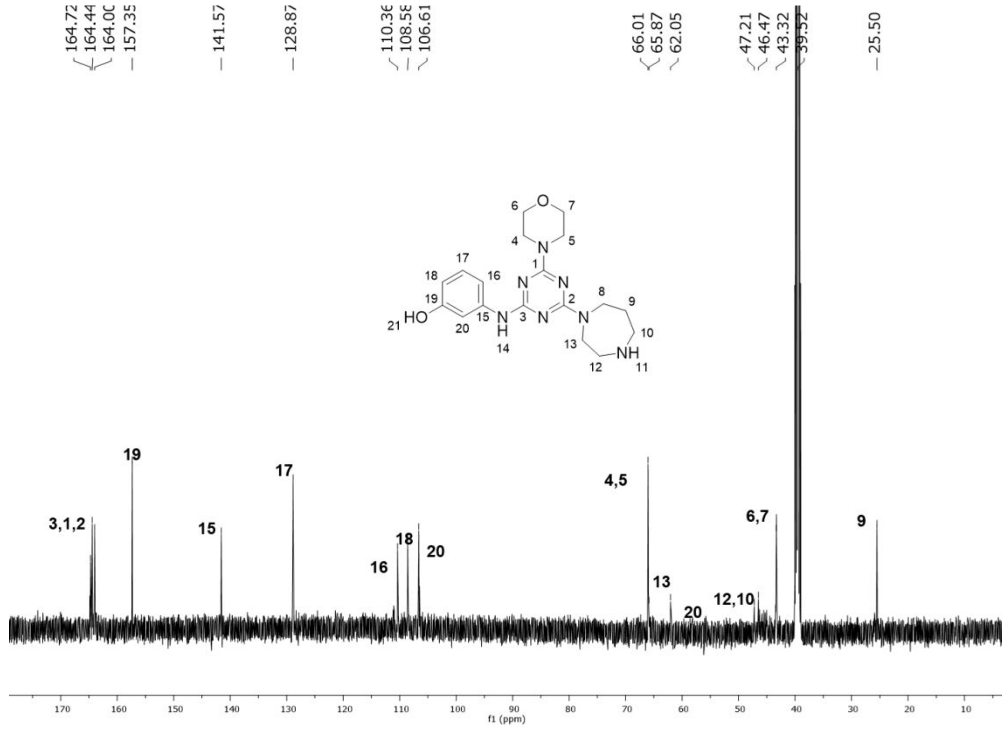

**Figure S7.** ***3-((4-(1,4-diazepan-1-yl)-6-morpholino-1,3,5-triazin-2-yl)amino)benzoic acid (9c)***

$^{1}$H-NMR

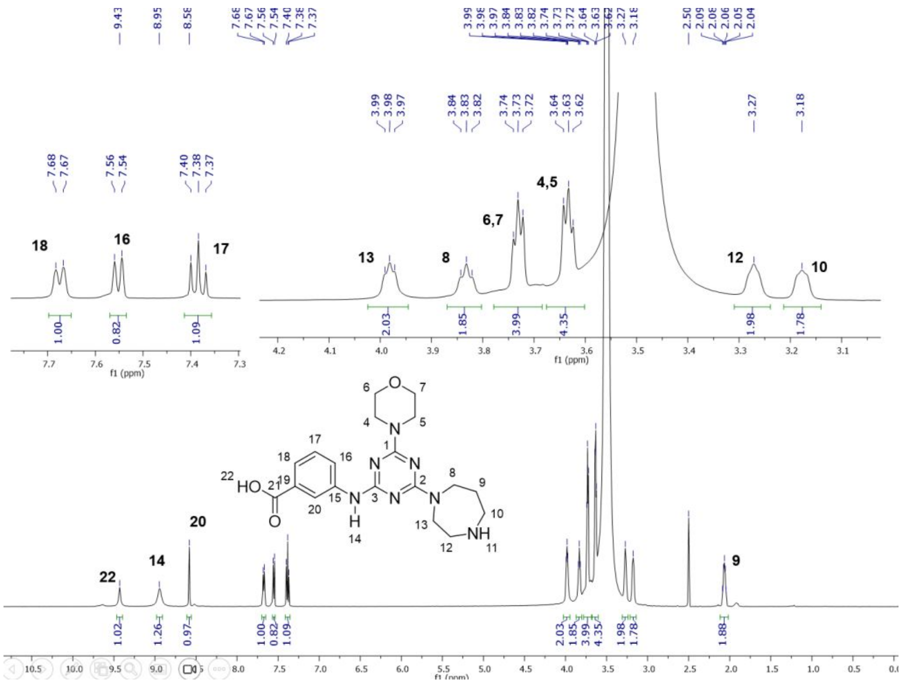


$^{13}$C-NMR

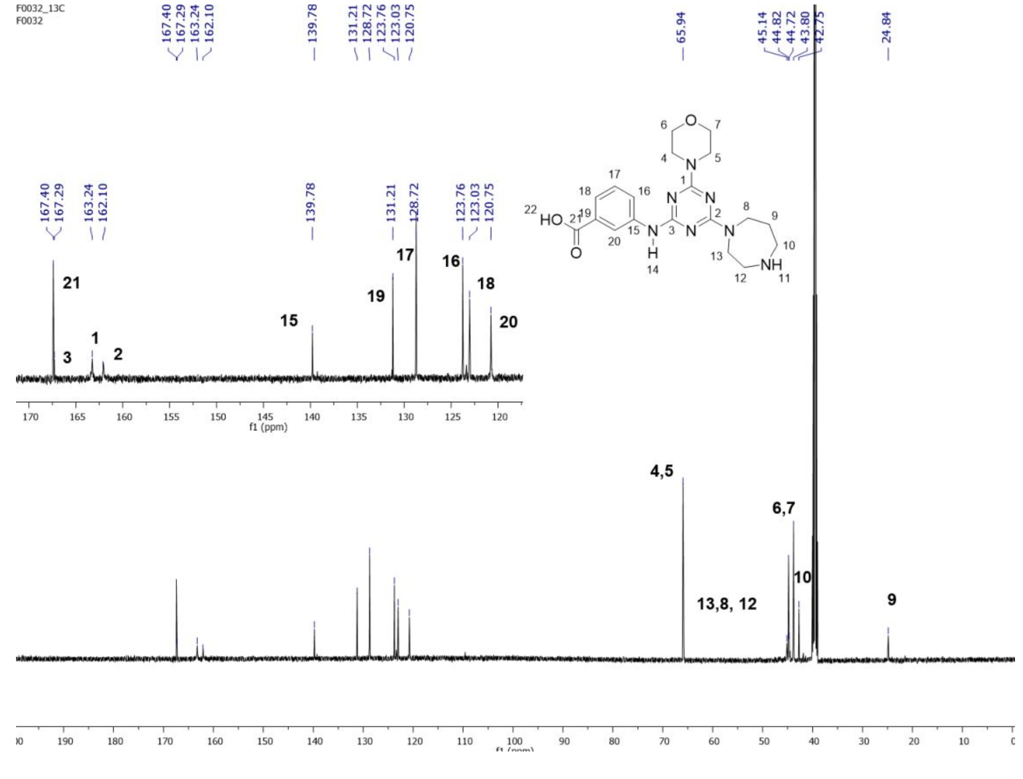

**Figure S8.** ***3-((4-(1,4-diazepan-1-yl)-6-morpholino-1,3,5-triazin-2-yl)amino)benzamide (9d)***

$^{1}$H-NMR

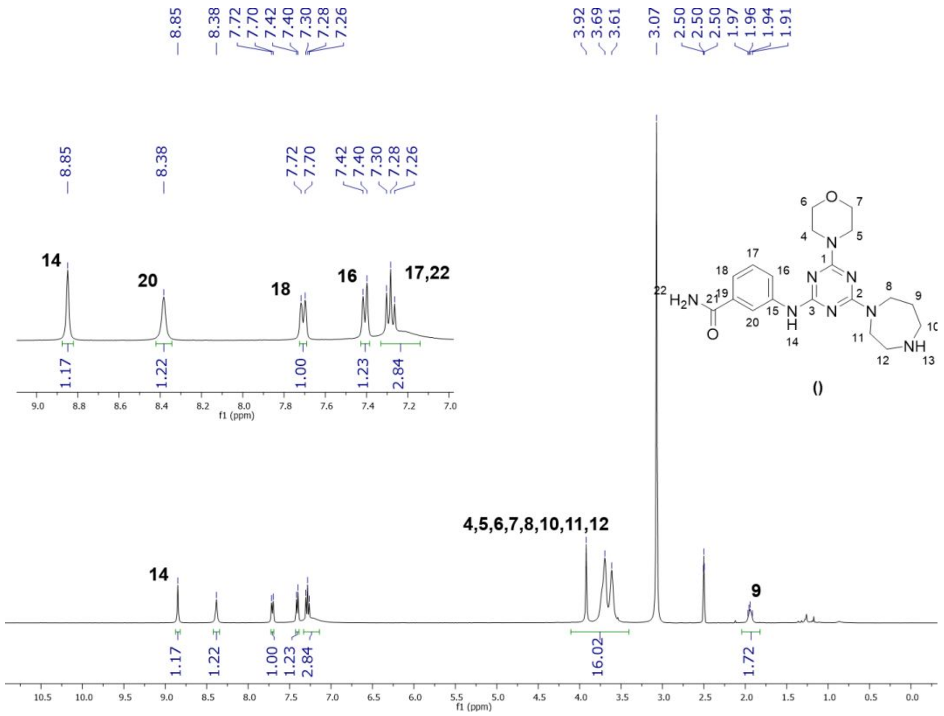


$^{13}$C-NMR

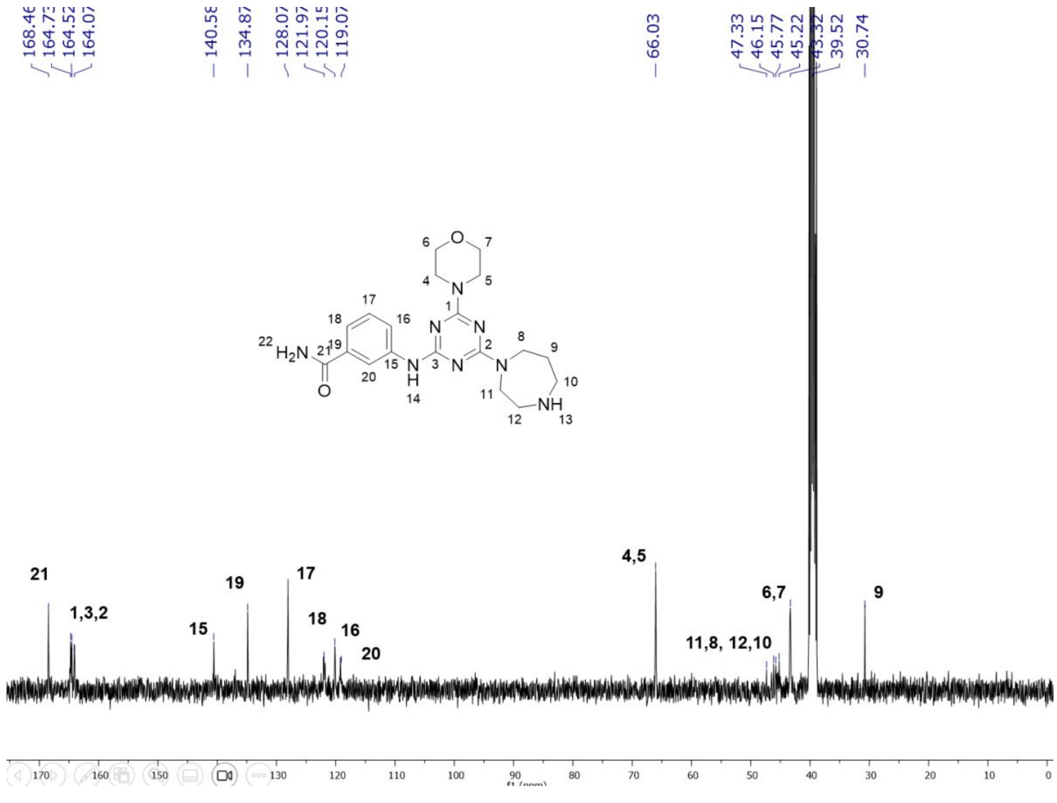

**Figure S9.** ***4-((4-(1,4-diazepan-1-yl)-6-morpholino-1,3,5-triazin-2-yl)amino)benzoic acid (9e)***

$^{1}$H-NMR

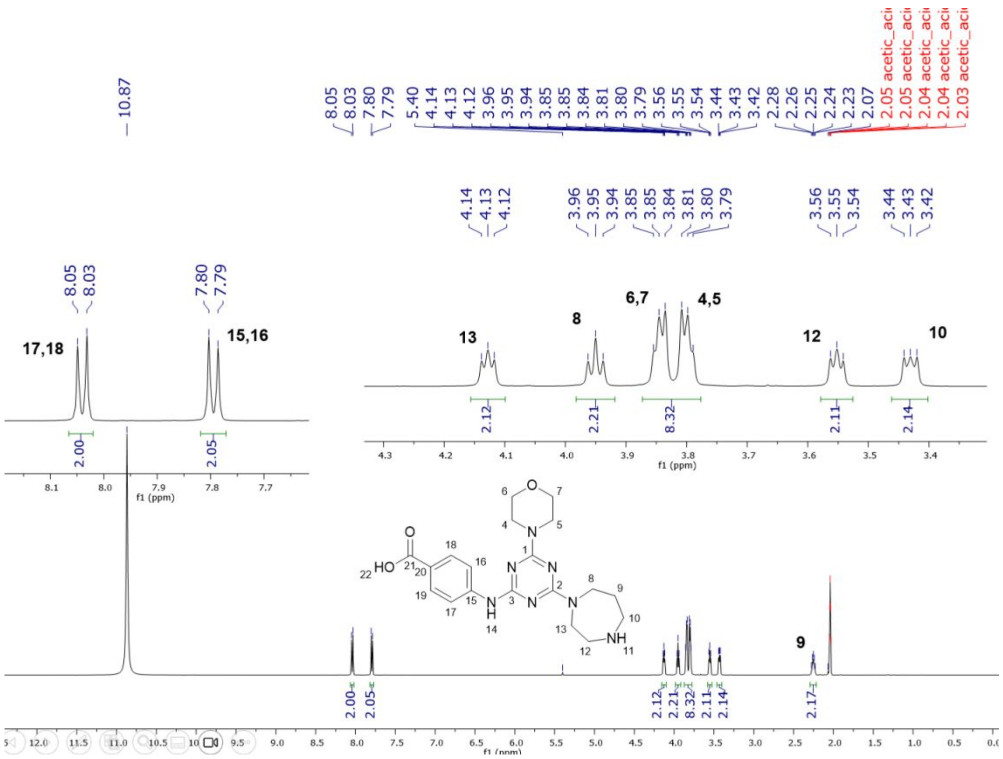

$^{13}$C-NMR

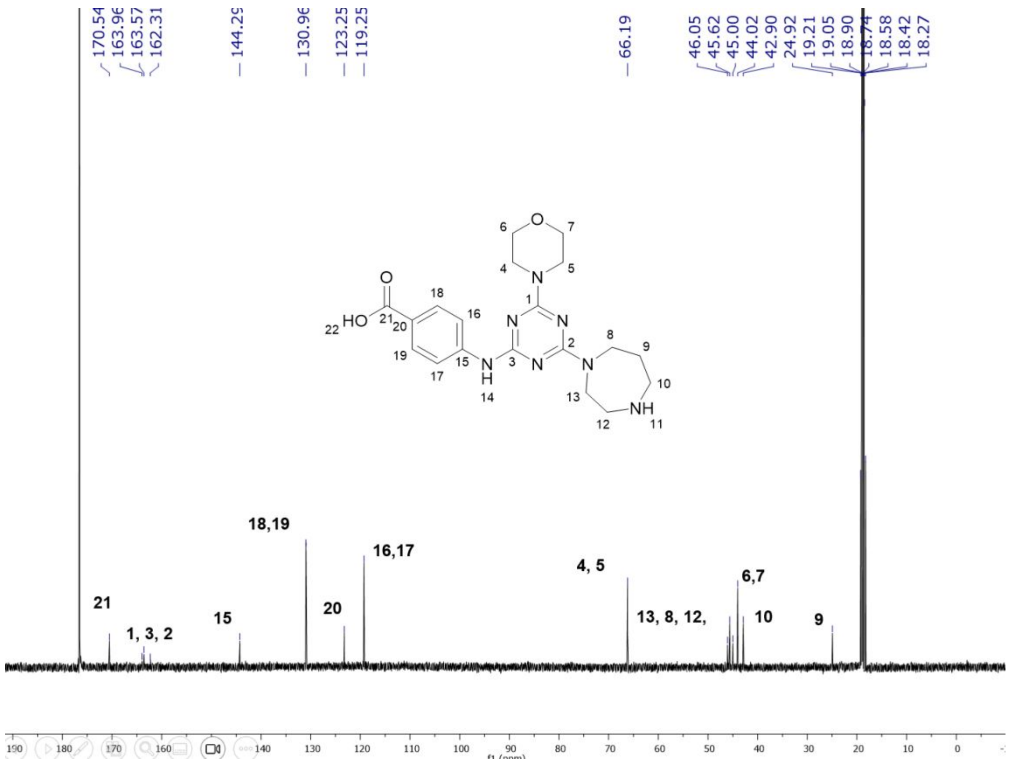

**Figure S10.** ***4-((4-(1,4-diazepan-1-yl)-6-morpholino-1,3,5-triazin-2-yl)amino)benzamide (9f)***

$^{1}$H-NMR

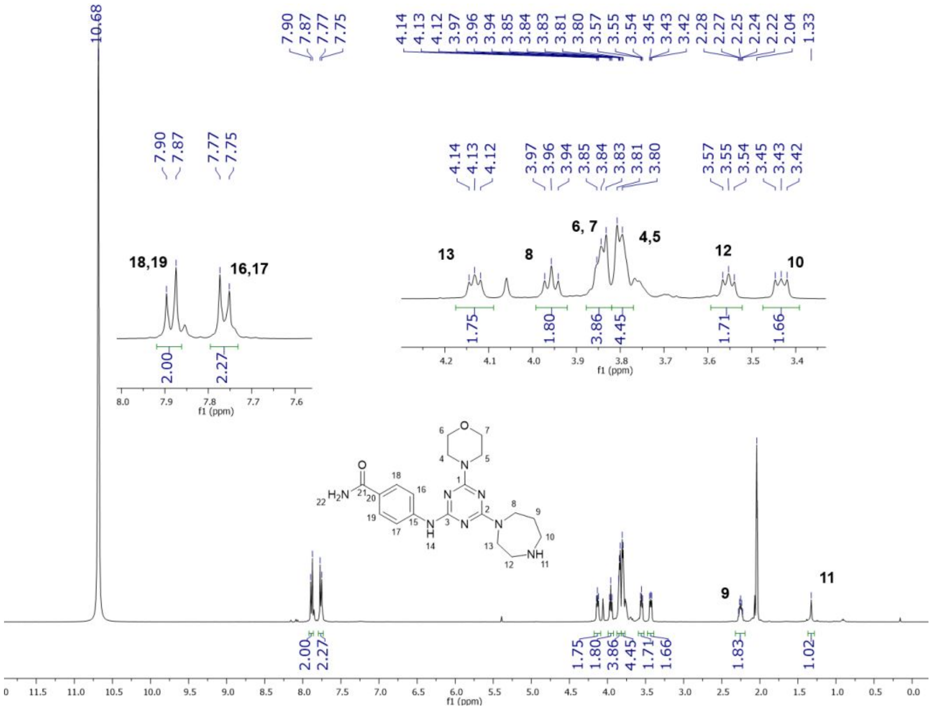


$^{13}$C-NMR

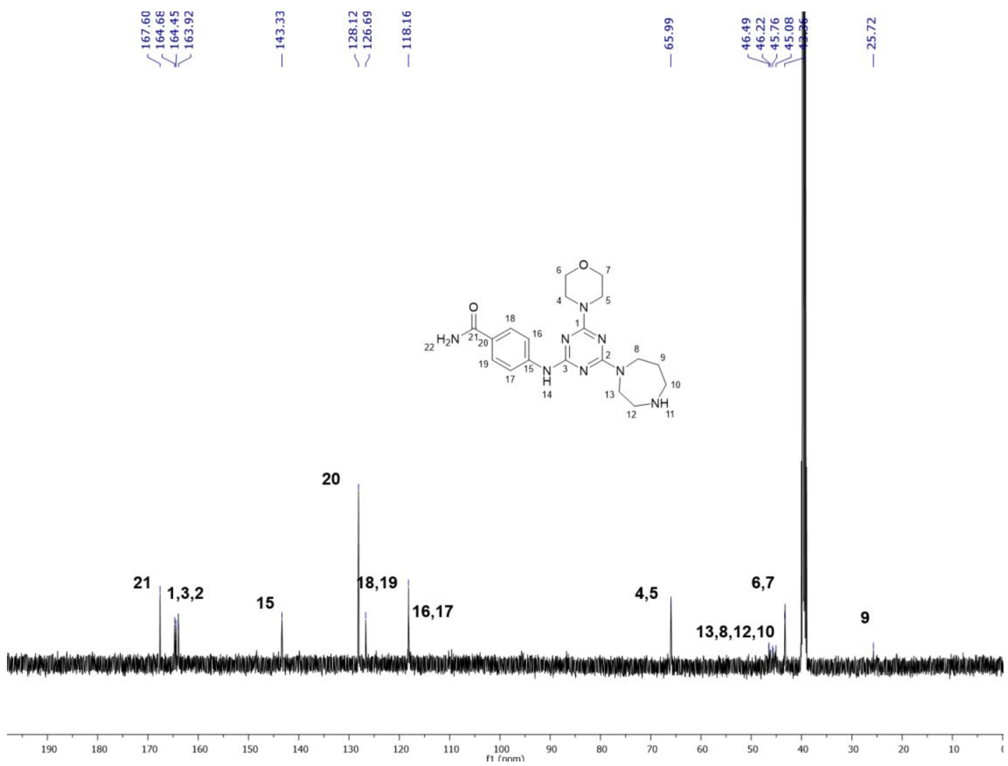

## HPLC TRACES OF FINAL COMPOUNDS

**Figure S11.** *3-(4-(1,4-diazepan-1-yl)-6-morpholino-1,3,5-triazin-2-yl) phenol (9a)*

### <Sample Information>

| | | | |
|---|---|---|---|
| Sample Name | : pf01 | | |
| Sample ID | : pf01 | | |
| Data Filename | : pf01.lcd | | |
| Method Filename | : 60MEOH(B)_254nm_15min.lcm | | |
| Batch Filename | : | | |
| Vial # | : 1-1 | Sample Type | : Unknown |
| Injection Volume | : 20 uL | | |
| Date Acquired | : 14/04/2023 11:05:06 | Acquired by | : System Administrator |
| Date Processed | : 14/04/2023 11:20:07 | Processed by | : System Administrator |

### <Chromatogram>

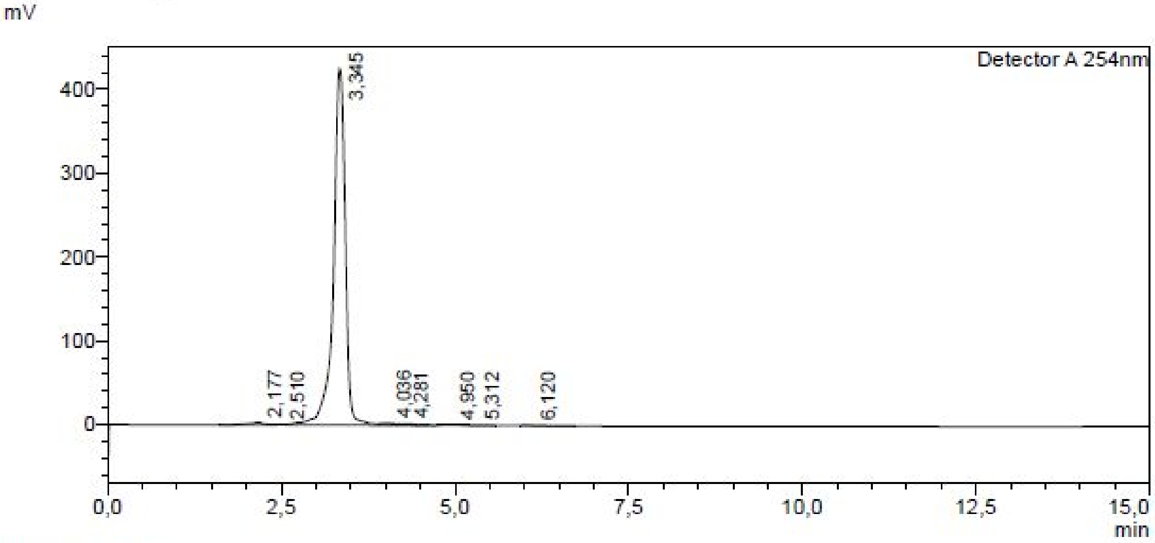


### <Peak Table>

Detector A 254nm

| Peak# | Ret. Time | Area | Height | Area% | Height% |
|---|---|---|---|---|---|
| 1 | 2,177 | 44430 | 3055 | 0,873 | 0,709 |
| 2 | 2,510 | 3712 | 518 | 0,073 | 0,120 |
| 3 | 3,345 | 5012905 | 425059 | 98,480 | 98,633 |
| 4 | 4,036 | 8955 | 846 | 0,176 | 0,196 |
| 5 | 4,281 | 11271 | 883 | 0,221 | 0,205 |
| 6 | 4,950 | 5250 | 384 | 0,103 | 0,089 |
| 7 | 5,312 | 1593 | 111 | 0,031 | 0,026 |
| 8 | 6,120 | 2137 | 94 | 0,042 | 0,022 |
| Total | | 5090252 | 430951 | 100,000 | 100,000 |

Detector B Ex:300nm,Em:400nm

| Peak# | Ret. Time | Area | Height | Area% | Height% |
|---|---|---|---|---|---|
| Total | | | | | |

**Figure S12.** ***3-((4-(1,4-diazepan-1-yl)-6-morpholino-1,3,5-triazin-2-yl)amino)phenol (9b)***

### <Sample Information>

| | | | |
|---|---|---|---|
| Sample Name | : pf02 | | |
| Sample ID | : pf02 | | |
| Data Filename | : pf02.lcd | | |
| Method Filename | : 60MEOH(B)_254nm_15min.lcm | | |
| Batch Filename | : | | |
| Vial # | : 1-1 | Sample Type | : Unknown |
| Injection Volume | : 20 uL | | |
| Date Acquired | : 14/04/2023 12:53:31 | Acquired by | : System Administrator |
| Date Processed | : 14/04/2023 13:08:32 | Processed by | : System Administrator |

### <Chromatogram>

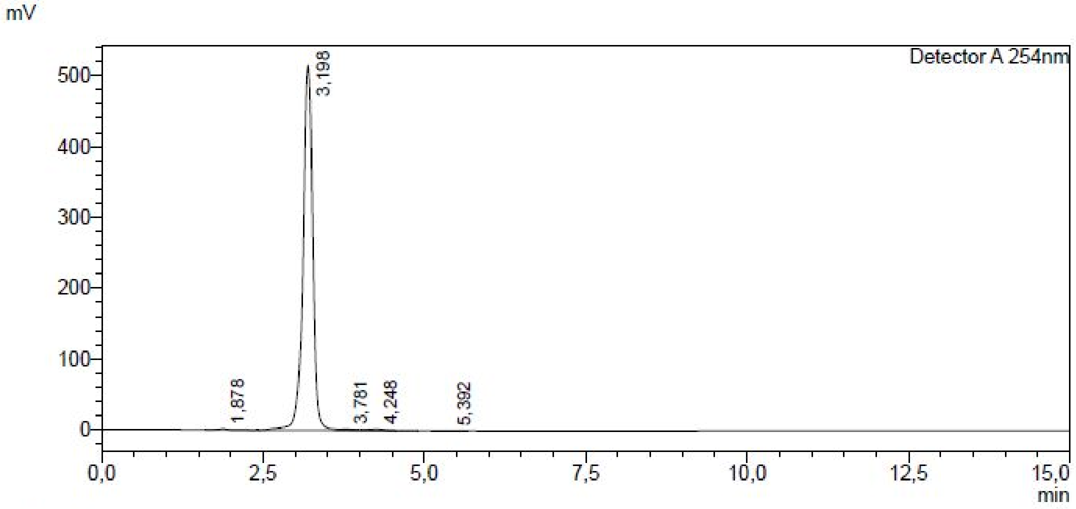


### <Peak Table>

Detector A 254nm

| Peak# | Ret. Time | Area | Height | Area% | Height% |
|---|---|---|---|---|---|
| 1 | 1,878 | 12242 | 1805 | 0,222 | 0,348 |
| 2 | 3,198 | 5475837 | 514085 | 99,231 | 99,179 |
| 3 | 3,781 | 5514 | 634 | 0,100 | 0,122 |
| 4 | 4,248 | 20255 | 1523 | 0,367 | 0,294 |
| 5 | 5,392 | 4443 | 293 | 0,081 | 0,056 |
| Total | | 5518291 | 518340 | 100,000 | 100,000 |

Detector B Ex:300nm,Em:400nm

| Peak# | Ret. Time | Area | Height | Area% | Height% |
|---|---|---|---|---|---|
| Total | | | | | |

**Figure S13.** *3-((4-(1,4-diazepan-1-yl)-6-morpholino-1,3,5-triazin-2-yl)amino)benzoic acid (9c)*

**<Sample Information>**

| | | | |
|---|---|---|---|
| Sample Name | : pf03-2004 | | |
| Sample ID | : pf03-2004 | | |
| Data Filename | : pf03-2005.lcd | | |
| Method Filename | : 60MEOH(B)_254nm_15min.lcm | | |
| Batch Filename | : | | |
| Vial # | : 1-1 | Sample Type | : Unknown |
| Injection Volume | : 20 uL | | |
| Date Acquired | : 20/04/2023 11:09:27 | Acquired by | : System Administrator |
| Date Processed | : 20/04/2023 11:24:28 | Processed by | : System Administrator |

**<Chromatogram>**

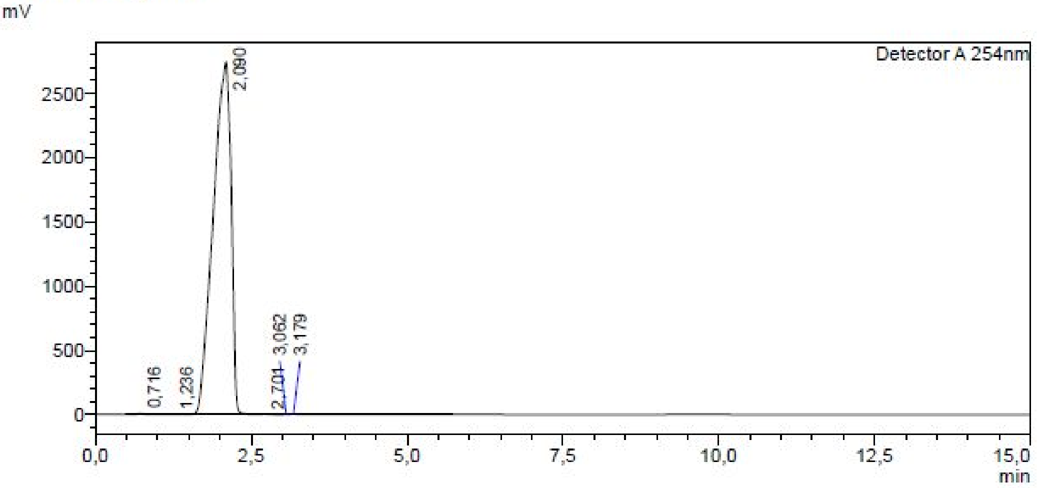


**<Peak Table>**

Detector A 254nm

| Peak# | Ret. Time | Area | Height | Area% | Height% |
|---|---|---|---|---|---|
| 1 | 0,716 | 58603 | 7088 | 0,107 | 0,257 |
| 2 | 1,236 | 2736 | 297 | 0,005 | 0,011 |
| 3 | 2,090 | 54769398 | 2742847 | 99,771 | 99,527 |
| 4 | 2,701 | 12814 | 1157 | 0,023 | 0,042 |
| 5 | 3,062 | 21273 | 2175 | 0,039 | 0,079 |
| 6 | 3,179 | 30359 | 2327 | 0,055 | 0,084 |
| Total | | 54895182 | 2755890 | 100,000 | 100,000 |

Detector B Ex:300nm,Em:400nm

| Peak# | Ret. Time | Area | Height | Area% | Height% |
|---|---|---|---|---|---|
| Total | | | | | |

**Figure S14.** *3-((4-(1,4-diazepan-1-yl)-6-morpholino-1,3,5-triazin-2-yl)amino)benzamide (9d)*

<Sample Information>

| | | | |
|---|---|---|---|
| Sample Name | : pf04 | | |
| Sample ID | : pf04 | | |
| Data Filename | : pf04.lcd | | |
| Method Filename | : 60MEOH(B)_254nm_15min.lcm | | |
| Batch Filename | : | | |
| Vial # | : 1-1 | Sample Type | : Unknown |
| Injection Volume | : 20 uL | | |
| Date Acquired | : 14/04/2023 13:35:14 | Acquired by | : System Administrator |
| Date Processed | : 14/04/2023 13:50:15 | Processed by | : System Administrator |

<Chromatogram>

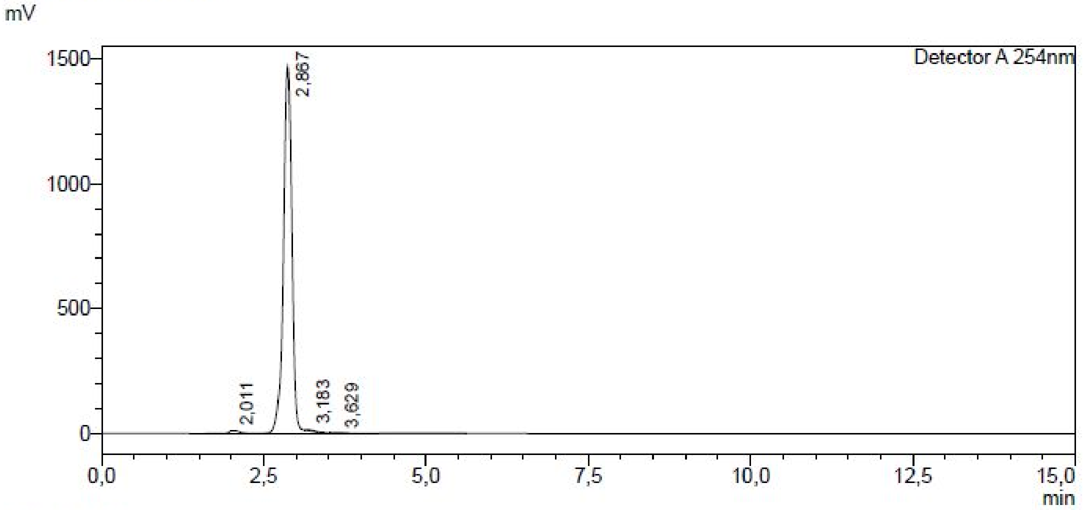


<Peak Table>

Detector A 254nm

| Peak# | Ret. Time | Area | Height | Area% | Height% |
|---|---|---|---|---|---|
| 1 | 2,011 | 149101 | 12345 | 1,087 | 0,830 |
| 2 | 2,867 | 13533858 | 1469423 | 98,632 | 98,814 |
| 3 | 3,183 | 28254 | 4040 | 0,206 | 0,272 |
| 4 | 3,629 | 10412 | 1253 | 0,076 | 0,084 |
| Total | | 13721625 | 1487061 | 100,000 | 100,000 |

Detector B Ex:300nm,Em:400nm

| Peak# | Ret. Time | Area | Height | Area% | Height% |
|---|---|---|---|---|---|
| Total | | | | | |

**Figure S15.** *4-((4-(1,4-diazepan-1-yl)-6-morpholino-1,3,5-triazin-2-yl)amino)benzoic acid (9e)*

**<Sample Information>**

Sample Name : pf05-2004
Sample ID : pf05-2004
Data Filename : pf05-2004.lcd
Method Filename : 60MEOH(B)_254nm_15min.lcm
Batch Filename :
Vial # : 1-1
Sample Type : Unknown
Injection Volume : 20 uL
Date Acquired : 20/04/2023 11:29:49
Acquired by : System Administrator
Date Processed : 20/04/2023 11:44:50
Processed by : System Administrator

**<Chromatogram>**

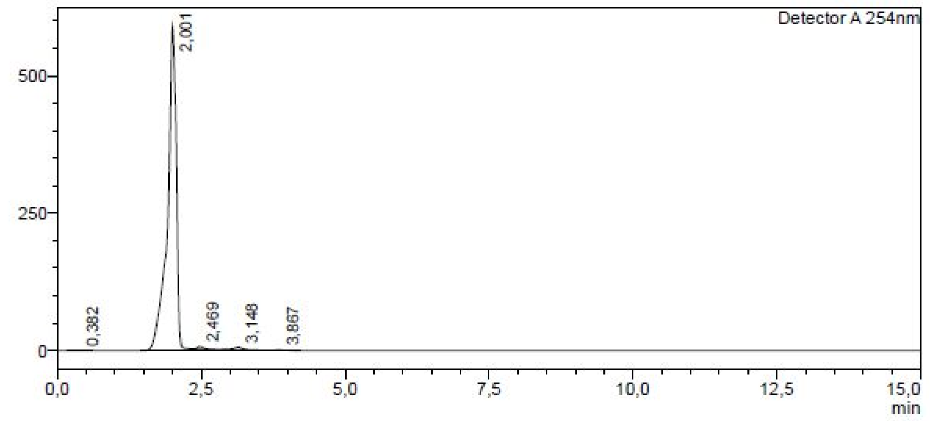


**<Peak Table>**

Detector A 254nm

| Peak# | Ret. Time | Area | Height | Area% | Height% |
|---|---|---|---|---|---|
| 1 | 0,382 | 1341 | 168 | 0,021 | 0,028 |
| 2 | 2,001 | 6396854 | 590841 | 98,410 | 98,260 |
| 3 | 2,469 | 38759 | 4931 | 0,596 | 0,820 |
| 4 | 3,148 | 59690 | 5070 | 0,918 | 0,843 |
| 5 | 3,867 | 3581 | 290 | 0,055 | 0,048 |
| Total | | 6500225 | 601302 | 100,000 | 100,000 |

Detector B Ex:300nm,Em:400nm

| Peak# | Ret. Time | Area | Height | Area% | Height% |
|---|---|---|---|---|---|
| Total | | | | | |

**Figure S16.** *4-((4-(1,4-diazepan-1-yl)-6-morpholino-1,3,5-triazin-2-yl)amino)benzamide (9f)*

<Sample Information>

Sample Name : pf06
Sample ID : pf06
Data Filename : pf06.lcd
Method Filename : 60MEOH(B)_254nm_15min.lcm
Batch Filename :
Vial # : 1-1
Injection Volume : 20 uL
Date Acquired : 14/04/2023 14:21:02
Date Processed : 14/04/2023 14:36:04

Sample Type : Unknown
Acquired by : System Administrator
Processed by : System Administrator

<Chromatogram>

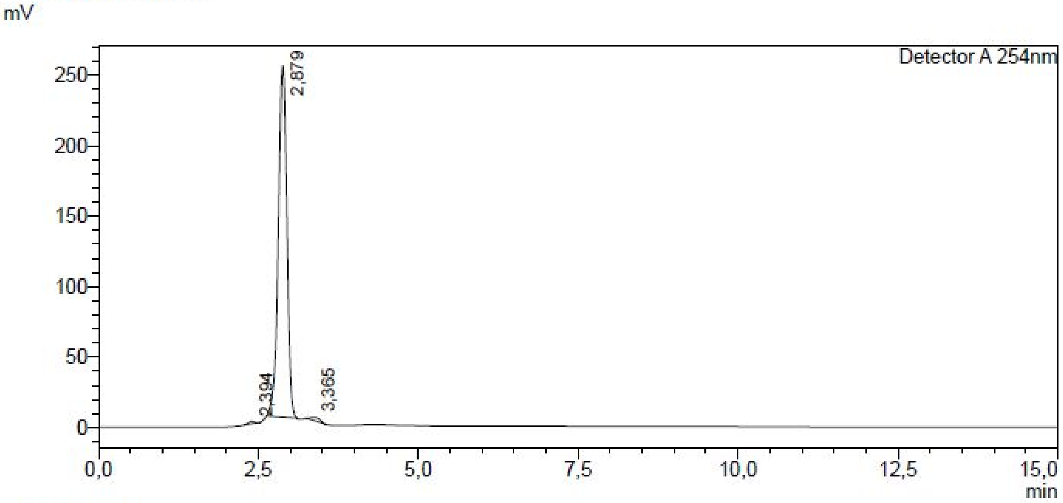


<Peak Table>

Detector A 254nm

| Peak# | Ret. Time | Area | Height | Area% | Height% |
|---|---|---|---|---|---|
| 1 | 2,394 | 10905 | 1686 | 0,466 | 0,667 |
| 2 | 2,879 | 2307081 | 248905 | 98,548 | 98,418 |
| 3 | 3,365 | 23086 | 2315 | 0,986 | 0,915 |
| Total | | 2341073 | 252906 | 100,000 | 100,000 |

Detector B Ex:300nm,Em:400nm

| Peak# | Ret. Time | Area | Height | Area% | Height% |
|---|---|---|---|---|---|
| Total | | | | | |

## MASS SPECTROMETRY DATA

**Figure S17.** ***Synthesis of 3-(4-(1,4-diazepan-1-yl)-6-morpholino-1,3,5-triazin-2-yl) phenol (9a)***

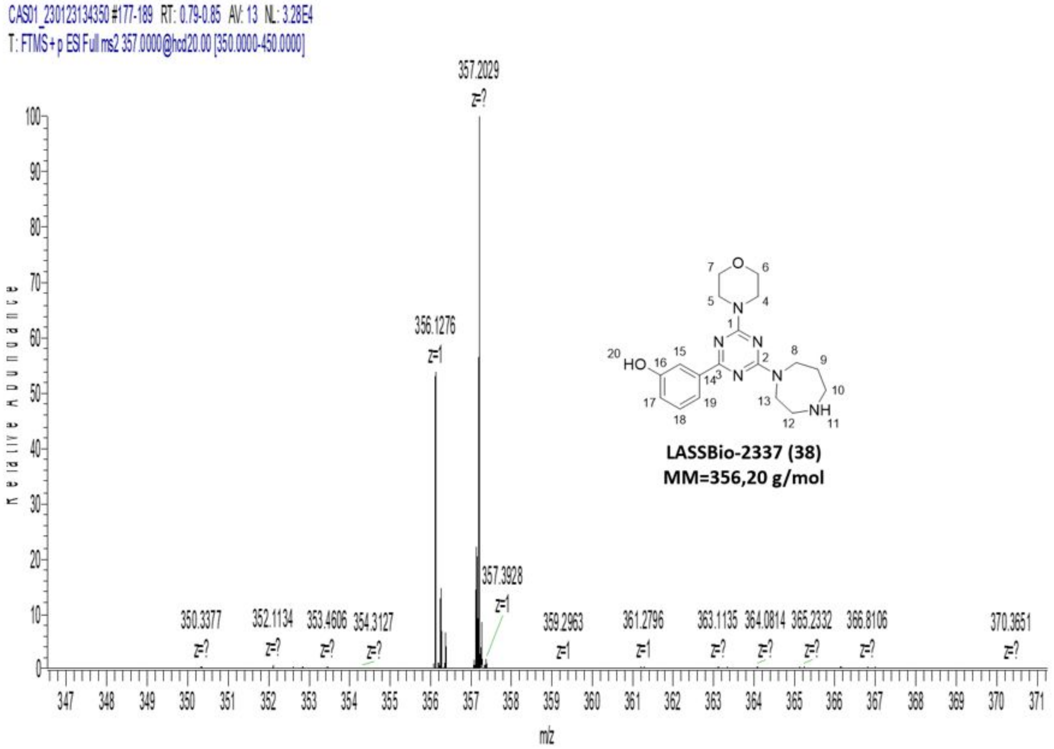


**Figure S18.** ***3-((4-(1,4-diazepan-1-yl)-6-morpholino-1,3,5-triazin-2-yl)amino)phenol (9b)***

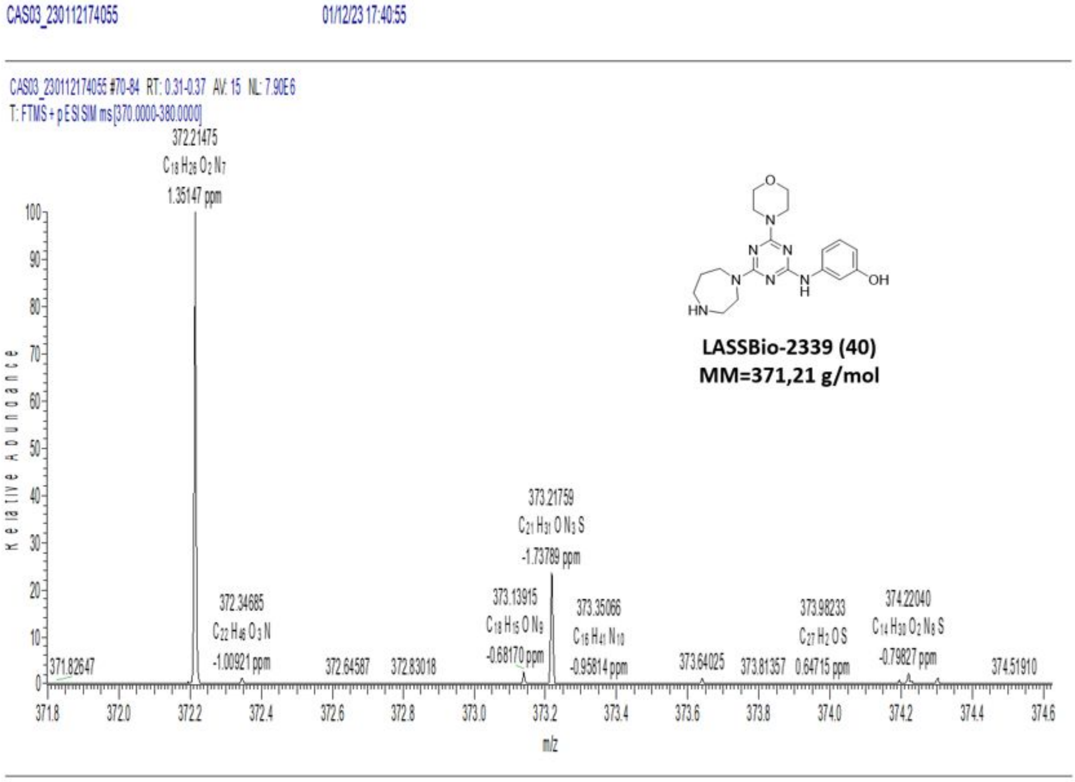

**Figure S19.** *3-((4-(1,4-diazepan-1-yl)-6-morpholino-1,3,5-triazin-2-yl)amino)benzoic acid (9c)*

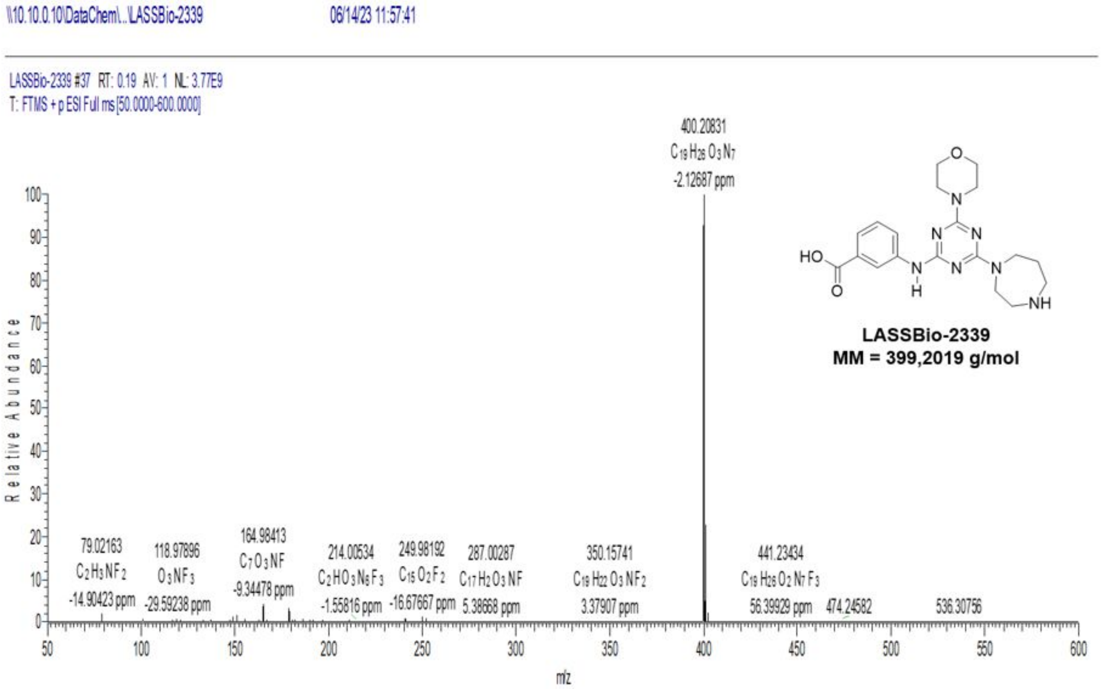


**Figure S20.** *3-((4-(1,4-diazepan-1-yl)-6-morpholino-1,3,5-triazin-2-yl)amino)benzamide (9d)*

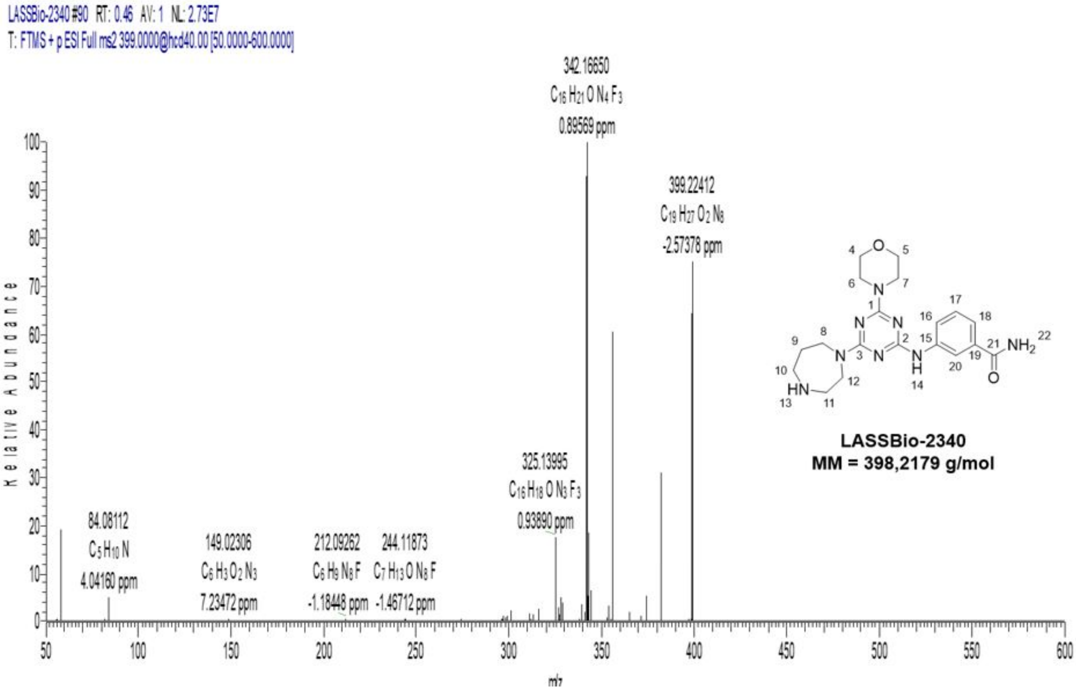

**Figure S21.** *4-((4-(1,4-diazepan-1-yl)-6-morpholino-1,3,5-triazin-2-yl)amino)benzoic acid (9e)*

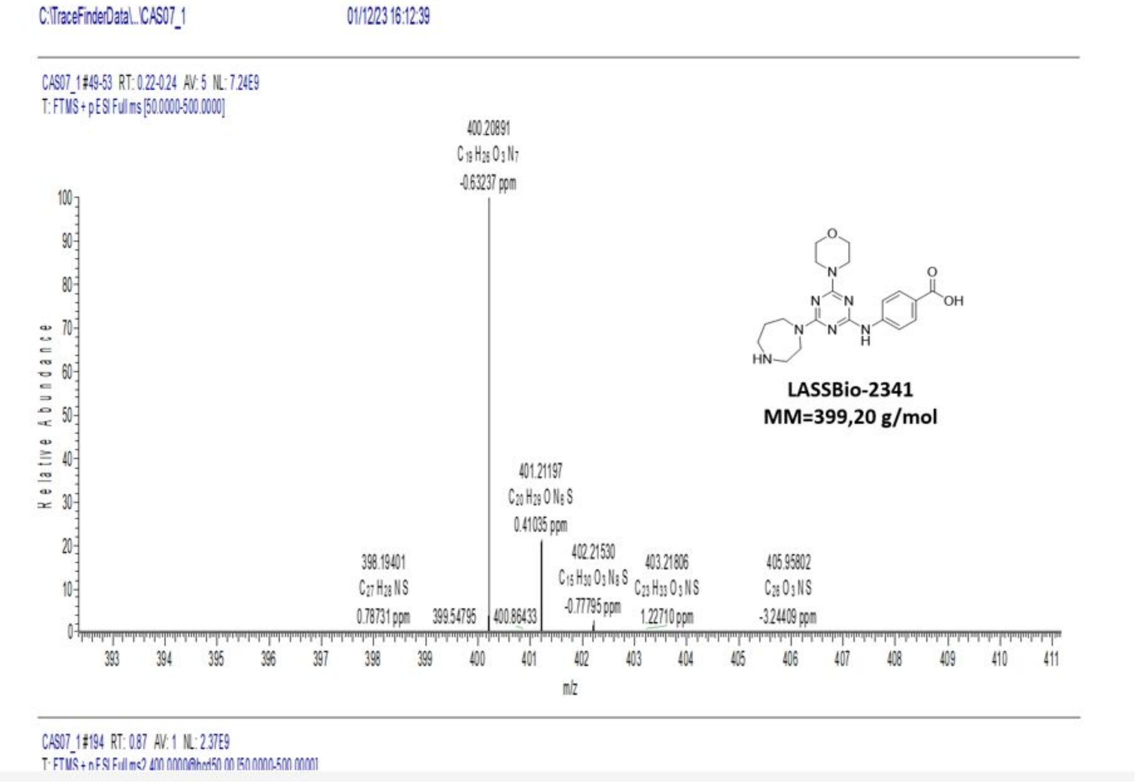

**Figure S22.** *4-((4-(1,4-diazepan-1-yl)-6-morpholino-1,3,5-triazin-2-yl)amino)benzamide (9f)*

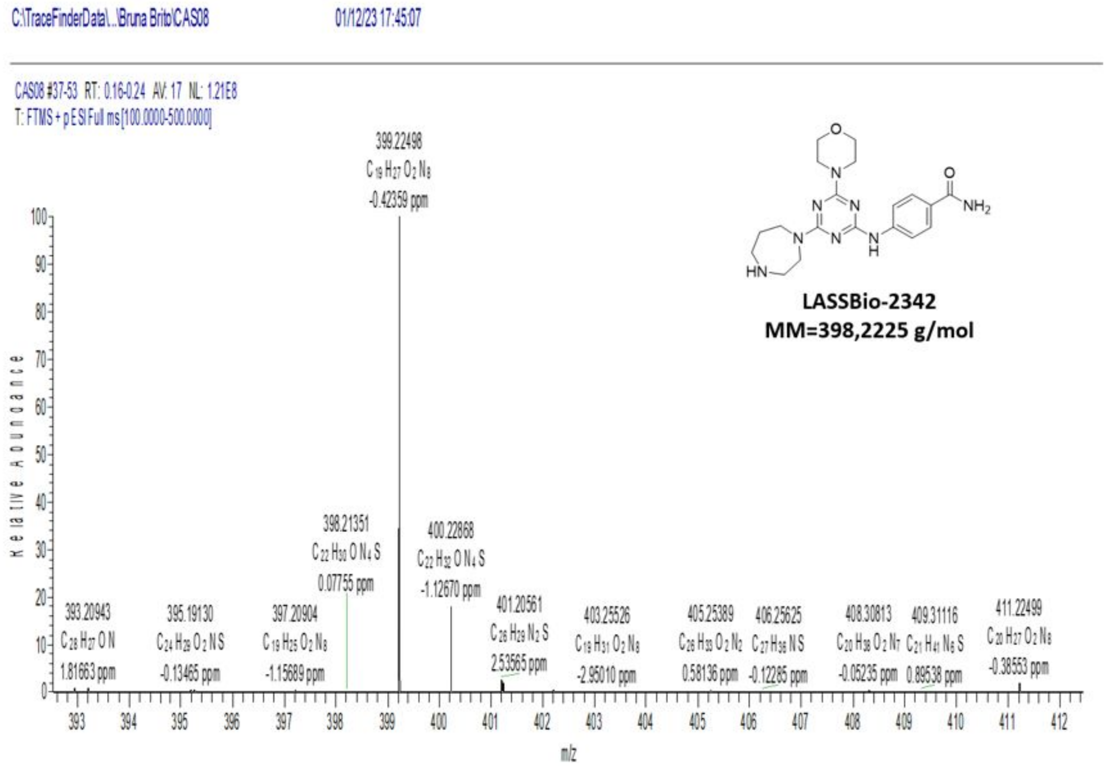

Supplement: Supplementary file 1 [file ao5c10162_si_001.pdf]
